# Spatio-temporal clustering of dengue in Valle del Cauca, Colombia

**DOI:** 10.1371/journal.pntd.0014855

**Published:** 2026-11-10

**Authors:** Hendrik Sy, Deninson A. Vargas-Gonzalez, Freddy Córdoba-Rentaría, Albert I. Ko, Johanna P. Daily, Neal D. Alexander

**Affiliations:** 1 Centro Internacional de Entrenamiento e Investigaciones Médicas (CIDEIM), Cali, Valle del Cauca, Colombia; 2 Division of Infectious Diseases, Albert Einstein College of Medicine, Bronx, New York, United States of America; 3 Universidad Icesi, Cali, Valle del Cauca, Colombia; 4 Secretaría de Salud del Valle del Cauca, Cali, Valle del Cauca, Colombia; 5 Yale School of Public Health, Yale University, New Haven, Connecticut, United States of America; 6 Instituto Gonçalo Moniz, Fundação Oswaldo Cruz/MS, Salvador, Bahia, Brazil; Universidade Federal de Minas Gerais, BRAZIL

## Abstract

**Background:**

Dengue is endemic in Colombia's Valle del Cauca region. The precise timing and spatial scale of seasonal dengue transmission necessary for effective reactive interventions at the sub-municipal level across the 42 municipalities of Valle del Cauca remain unclear. This study aimed to identify spatio-temporal dengue clusters in Valle del Cauca.

**Methodology/Principal Findings:**

A retrospective observational study was conducted using individual laboratory-confirmed dengue cases, including residential geolocation and consultation dates, reported by healthcare providers to the Sistema Nacional de Vigilancia en Salud Pública (SIVIGILA) in 2022. Residence was utilized as the probable site of infection. Spatio-temporal clustering was analyzed using Kulldorff's space-time scan statistic to identify statistically significant dengue clusters. Results were mapped using R. As a second aim, the association between clinical and demographic characteristics and dengue hospitalization was evaluated. Of 3,964 dengue cases reported in Valle del Cauca in 2022, 2,232 (56%) were laboratory-confirmed. Of these, 1,973 occurred in the defined areas of the nine municipalities included in the spatio-temporal analysis of this study. We identified 28 clusters at the sub-municipal level, with radii of 0–0.98 km, lasting 1–6 months and containing 10–114 cases. Twenty-six clusters (92.9%) spanned both warm–dry and cool–wet seasons, and clusters accounted for 32.0% of spatially analyzed cases. 960 patients were hospitalized. Lower socioeconomic status (OR 1.30, 95% CI 1.07–1.57), enrollment in the state-subsidized insurance scheme (OR 1.74, 95% CI 1.41–2.15) compared with the contributory scheme, and age ≥ 70 years compared to ages 20–29 years (OR 2.47, 95% CI 1.51–4.04) were associated with higher odds of hospitalization.

**Conclusions/Significance:**

This study identified dengue clusters at the sub-municipal level with the potential to inform reactive dengue control interventions and demonstrated a disproportionate impact on socioeconomically disadvantaged populations and the elderly in Valle del Cauca, Colombia.

## Author summary

Dengue is a common cause of febrile illness in many tropical regions, including the Colombian department of Valle del Cauca. We wanted to understand where and when dengue cases group together within cities, and which patients are more likely to be admitted to the hospital. We used routine health surveillance data from nine municipalities in 2022 to map areas that had more dengue cases than expected over space and time. We found dengue “hotspots”, areas with radii of less than one kilometer, where reported cases clustered over several months and across different seasons. We also found that people living in low-income neighborhoods, those without insurance or with a state-subsidized insurance scheme, and older adults were more likely to be hospitalized with dengue. These findings suggest that dengue control could be made more efficient by focusing mosquito control and community actions on hotspot areas, and providing timely diagnosis and improving health care access for lower income residents and older adults.

## Introduction

Timely information on where and when dengue transmission occurs within urban areas is critical for planning reactive vector control interventions. However, the spatial scale, duration, and seasonal timing of sub-municipal dengue clusters that could serve as operational targets remain poorly characterized in many endemic settings.

In Valle del Cauca, dengue is endemic and recurrent epidemics require timely information on where transmission is occurring. National dengue incidence has fluctuated substantially, with epidemic cycles in 2010, 2013, 2016, 2019, and 2023 [1]. Dengue risk is geographically heterogeneous, and the Colombian Instituto Nacional de Salud (INS) classifies high-risk municipalities using criteria that include altitude, *Aedes aegypti* presence, prior dengue cases, and sustained transmission [2]. The record dengue burden reported in the Americas in 2024 further supports the need to identify local transmission hotspots that can guide control activities [3].

Dengue virus (DENV) is transmitted by *Aedes aegypti* and *Aedes albopictus* mosquitoes, and its transmission dynamics are influenced by climatic conditions, vector abundance and vectorial competence, circulating serotypes, social determinants, and population density [4]. In Colombia, dengue is a nationally reportable disease, and healthcare providers must report cases to the Sistema Nacional de Vigilancia en Salud Pública (SIVIGILA) [1,5]. Vector control remains the primary strategy for dengue prevention. The World Health Organization (WHO) recommends elimination of mosquito breeding sites, using insecticides or biological agents, and adapting interventions based on local contexts and available resources [6]. In Valle del Cauca, control measures are coordinated by the Secretaría de Salud del Valle del Cauca. However, targeted reactive interventions for control of dengue outbreaks require granular, location-specific data on high-risk transmission sites for dengue and individual-level risk factors, which remain underexplored at the sub-municipal level in Colombia.

Geospatial analysis techniques, such as Kulldorff’s space-time scan statistic, have been used to identify spatial and temporal clusters of dengue cases, improving surveillance and control strategies [7–13]. Prior studies have demonstrated the value of these methods in endemic regions for dengue, but few have applied them at sub-municipal scales in Colombia [8,13]. Given the limited resources in low- and middle-income countries (LMICs) and the fact that dengue transmission is influenced by local factors below the municipal level [14], identifying precise clustering of dengue infections could enhance targeted vector control interventions by health authorities. Prior studies have examined the spatial epidemiology of dengue and other arboviruses in Colombia, including in Cali. Earlier analyses described fine-scale spatio-temporal patterns of dengue in Cali [15], and modeled socioeconomic and environmental determinants of dengue risk at the neighborhood level [14,16]. More recent studies have applied space–time conditional autoregressive (CAR) models to estimate neighborhood-level dengue risk [17], and have characterized space–time clusters and co-occurrence of chikungunya and dengue across Colombia [18], while complementary work has documented community knowledge, attitudes, and practices and engaged public health stakeholders to inform surveillance design [19–21]. A systematic review further situates these findings within structural inequities driving Aedes-borne disease [22]. Our analysis differs in four respects: (i) cluster size is operationally constrained to a sub-kilometer scale to identify areas small enough for targeted vector-control activities, (ii) analytical parameters were co-defined with the Secretaría de Salud to support local public health use, (iii) the primary cluster analysis is restricted to laboratory-confirmed cases to limit misclassification, and (iv) cluster detection is combined with patient-level modeling of dengue-related hospitalization.

This study had two aims: 1) to retrospectively identify spatio-temporal clusters of laboratory-confirmed dengue cases below the municipal level in Valle del Cauca, Colombia, in 2022 and 2) to assess the association between clinical and socio-demographic characteristics and hospitalization from dengue, to guide resource allocation for reactive vector control interventions and inform risk stratification for the management of patients with dengue. We hypothesized that laboratory-confirmed dengue cases would show localized clustering in space and time at the sub-municipal level, and that dengue-related hospitalization would vary by socioeconomic stratum.

## Methods

### Ethical considerations

This study was approved by the Institutional Review Board (IRB) at Centro Internacional de Entrenamiento e Investigaciones Médicas (CIDEIM) and the Secretaría de Salud del Valle del Cauca, Cali, Colombia and of Albert Einstein College of Medicine, Bronx, United States of America. The study was conducted in compliance with the Declaration of Helsinki (2013 revision), the CIOMS guidelines (2016 revision), the Good Clinical Practice (ICH-GCP) guidelines, and Colombian regulations (Resolution 008430 of 1993).

### Study design and data collection

A retrospective observational study of dengue cases was conducted using the SIVIGILA database provided by the Secretaría de Salud del Valle del Cauca. It contains healthcare provider-reported dengue case data for the *departamento* (“department”) of Valle del Cauca in 2022. To conduct a spatio-temporal cluster analysis of laboratory-confirmed dengue cases, nine municipalities at high and very high risk for dengue transmission in Valle del Cauca were prioritized based on criteria of the Colombian INS [2]. The INS defines high-risk areas as municipalities below 2,200 meters above sea level with a recorded presence of *Aedes aegypti*, a history of dengue cases, and sustained transmission over a six-year period using a point system. The prioritized municipalities were Yumbo, Cartago, Tuluá, Buga, Palmira, Florida, Candelaria, Jamundí, and Cali (Fig 1).

**Fig 1:**
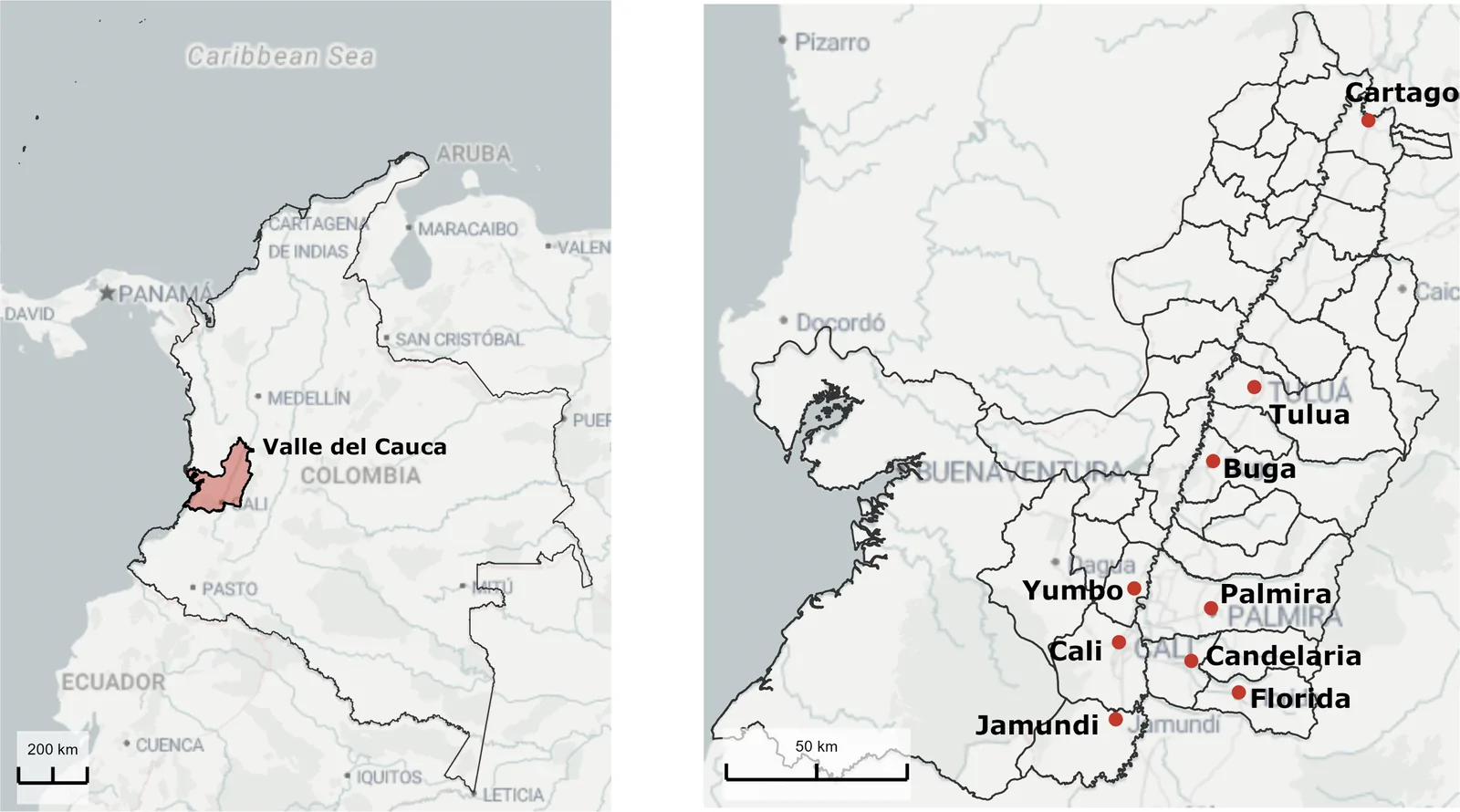
Location of Valle del Cauca and municipalities included in spatio-temporal analysis. Map of Colombia with department of Valle del Cauca in the Southwest Pacific region (left). Map of Valle del Cauca with nine municipalities at high- or very high risk for dengue transmission included in the spatio-temporal analysis of dengue clusters (right). Administrative boundaries were obtained from the GADM Database of Global Administrative Areas, version 4.1, Colombia administrative levels 0–2. Direct boundary dataset: https://geodata.ucdavis.edu/gadm/gadm4.1/shp/gadm41_COL_shp.zip. Basemap tiles Stadia Maps; Stamen Design (CC BY 4.0), OpenMapTiles; OpenStreetMap contributors (ODbL). See stadiamaps.com/attribution, openmaptiles.org, openstreetmap.org.

For the spatio-temporal analysis in Aim 1), residential addresses and consultation date of dengue patients were collected from the SIVIGILA database. For the risk factor analysis in Aim 2), hospitalization, socioeconomic stratum, age, sex, ethnicity, healthcare insurance scheme, dengue severity, and clinical care setting were collected from the SIVIGILA database.

In Colombia, reporting of dengue cases is mandatory for both public and private healthcare facilities. Probable dengue cases are defined by the INS and are confirmed through laboratory testing or epidemiological linkage [1]. The primary analyses included only laboratory-confirmed dengue cases. The hospitalization sensitivity analysis included all notified cases irrespective of laboratory confirmation. For laboratory confirmation, the INS uses dengue non-structural protein 1 (NS1) testing by enzyme-linked immunosorbent assay (ELISA), reverse transcription polymerase chain reaction (RT-PCR), or viral isolation in patients within 5 days from symptom onset, or immunoglobulin M (IgM) ELISA in patients with 6 or more days since symptom onset [1]. Patients’ residential addresses were used as a proxy for the probable site of dengue infection. Data from all ages were included.

### Study area

The department of Valle del Cauca is located in southwestern Colombia and has a tropical climate with two main seasons: warm-dry periods from December to February and June to September, and cool-wet periods from March to May and October to November [23]. Cali, the department’s capital city, is situated in the valley of the River Cauca at 1,018 meters above sea level with a population of 2,227,642 in a 2018 census [24]. During warm-dry seasons Cali experiences average maximum temperatures of 32°C, with 54.6 mm of precipitation monthly. During cool-wet seasons, maximum temperatures drop to 28°C, and monthly rainfall increases to 107.4 mm. Other cities in the Cauca River valley, such as Palmira (at 1,001 meters, population: 361,375), Buga (at 969 meters, population: 128,945), and Jamundí (at 869 meters, population: 124,586), experience similar climatic patterns [23,24]. Valle del Cauca was selected for its high dengue incidence and our established partnership with the Secretaría de Salud, which facilitates rapid translation of findings into public health practice.

### Spatio-temporal analysis

Residential addresses were converted to latitude and longitude coordinates with QGIS (version 3.32) using the WGS84 coordinate system before analysis. A population raster from an open-source dataset was used to derive population estimates from United Nations data at an approximately 100 m × 100 m resolution [25]. Dengue clusters were identified with the Kulldorff method using SaTScan [26,27]. This method applies a space-time scan statistic with a cylindrical scanning window with a circular geographic base to detect statistically significant dengue clusters. The model assumed a discrete Poisson distribution. Cases were selected within predefined latitude–longitude rectangles that encompassed the urban areas in the prioritized municipalities. The population raster was cropped to the bounding extent of the selected case points, retaining its grid. Each study area with eligible cases was scanned separately using a spatial window of 20% of the population at risk and a maximal cluster radius of 1 km. These parameters were selected based on discussions with stakeholders from the Secretaría de Salud. Smaller cluster sizes were considered more practical for identifying high-risk sites and optimizing vector control resource allocation. The minimum cluster size was 10 cases. The maximum temporal window was set at 50% of the study period (6 months). This upper bound allowed shorter clusters while evaluating elevated risk over several months. Sensitivity analyses used 3- and 1-month maxima with the other settings unchanged. Significant clusters were determined using Monte Carlo simulations (9,999 replications, *p* < 0.05). Spatial visualization was performed using R, and cluster characteristics, including cluster radius, relative risk (RR), cases and population in the detected cluster, were documented. These parameters were compared between temporal maxima in the sensitivity analysis. The RR indicates how much more likely cases are to occur within the detected cluster than in the remainder of the study area and study period. Each case retained its consultation date recorded in SIVIGILA. Counts were summed by raster cell and date before monthly aggregation. The choice of spatial and temporal windows reflected methodological convention and operational constraints. The 20% upper bound on the population at risk and the maximum cluster radius of 1 km were selected to support an operational signal for reactive vector control measures at the neighborhood level. All analyses were performed using R software (package: terra) and SaTScan (version 10.3.2).

### Risk factor analysis for hospitalization among dengue patients

To evaluate the association between clinical and sociodemographic variables and hospitalization among patients with laboratory-confirmed dengue in Valle del Cauca in 2022, dengue cases from the SIVIGILA database were analyzed.

Variables were assessed for missingness, and variables with less than 10% missing data were retained. Data with missing variables were excluded. Hospitalization (yes/no) was used as the dependent variable, while socioeconomic stratum (SES) was defined as the independent variable of interest, dichotomized into low (SES 1–2) and middle-high (SES 3–6). In Colombia, the National Administrative Department of Statistics (DANE) determines SES based on the classification of residential properties, using physical and economic characteristics of the housing and its surrounding environment to determine contributions for public services. The range is from 1, representing the lowest, to 6 representing the highest socioeconomic stratum [28]. SES was dichotomized as low, defined as strata 1–2, versus middle or higher, defined as strata 3–6, for analytic reasons. In Colombia, socioeconomic stratification is an administrative classification of residential properties used primarily for differential billing of public utilities, including subsidies [29,30]. DANE classifies strata 1 and 2 as “low-low” and “low,” while stratum 3 is classified as “medium-low” [29]. Although stratum 3 may be eligible for public-utility subsidies under certain regulatory conditions [30], strata 4–6 were sparse in our dataset. We therefore combined strata 3–6 to provide a stable comparator group. Prior Colombian dengue research has defined strata 1–2 as low SES, although some studies used three-level groupings separating strata 3–4 and 5–6 [31].

To evaluate the association between the independent variables and hospitalization, a multivariable logistic regression model was employed. The following independent variables from SIVIGILA were considered, as applicable: socioeconomic stratum (low vs. middle-high SES), age, sex, ethnicity (Black/Afro-Colombian vs. other), healthcare coverage (contributory, subsidized, uninsured, other), dengue severity (dengue without warning signs, dengue with warning signs, and severe dengue), and clinical care setting (ambulatory care, general floor, intensive care unit, observation unit, referral to hospital).

Bivariate analyses were conducted as appropriate for continuous and categorical variables. Statistical assumptions were assessed for all analyses performed in this study. Variables were selected for model construction based on their significance in bivariate analyses (p ≤ 0.25) or their clinical relevance. Exploratory first-order interactions between socioeconomic stratum and the other model variables were evaluated when estimable using a significance level of α = 0.05. Confounding was assessed using a 15% change in the β coefficient for the variable of interest socioeconomic stratum, which represents its association with hospitalization, when each covariate was removed individually. Multicollinearity among variables was evaluated using the variance inflation factor (VIF). Model goodness-of-fit was assessed using the Hosmer-Lemeshow test. Model specification was assessed using the link-test squared term. Statistical analyses assumed a significance level of p < 0.05, and odds ratios (OR) with 95% confidence intervals were reported to quantify associations between the independent variables and hospitalization. Lastly, we conducted bivariate analyses comparing laboratory-confirmed and non–laboratory-confirmed cases, and performed a sensitivity analysis of the multivariable logistic regression model including all notified dengue cases irrespective of laboratory confirmation status to assess the robustness of the dengue case definition. All analyses were performed using Stata (version 18.0).

Sample size calculations: We used dengue surveillance data, so the sample size was determined by the number of eligible notified cases during the study period. To estimate the sample size required for an assumed effect size for the primary socioeconomic exposure (low SES strata 1–2 vs strata 3–6) among laboratory-confirmed cases, we performed a retrospective sample-size calculation using a two-sample comparison of proportions with unequal group sizes with two-sided α = 0.05. Using the observed prevalence of low SES (px = 0.593, 1,248/2,104) and the observed hospitalization probability in the reference group (medium–high SES, p0 = 0.389), we specified an effect size of OR=1.30, corresponding to an implied hospitalization risk of p1 = 0.453 in the low-SES group. Under these assumptions, the required sample size for 80% power was n = 1,947. The final analytical dataset included 2,104 laboratory-confirmed cases with non-missing SES and hospitalization data (856 medium–high SES, 1,248 low SES), which met this requirement.

## Results

### Spatio-temporal clustering of dengue cases

In 2022, 3,964 dengue cases were recorded in Valle del Cauca and 2,232 were laboratory-confirmed cases (56%) (Fig 2). For the spatio-temporal analysis of dengue clusters, 1,973 laboratory-confirmed cases (50% of all notified cases) were included, which had recorded street addresses and were within the defined study areas of the nine municipalities chosen for analysis of Aim 1 because they were classified as high risk for dengue transmission by the INS and were prioritized by the Secretaría de Salud. All 2,232 laboratory-confirmed cases were considered for the risk factor analysis of Aim 2.

**Fig 2:**
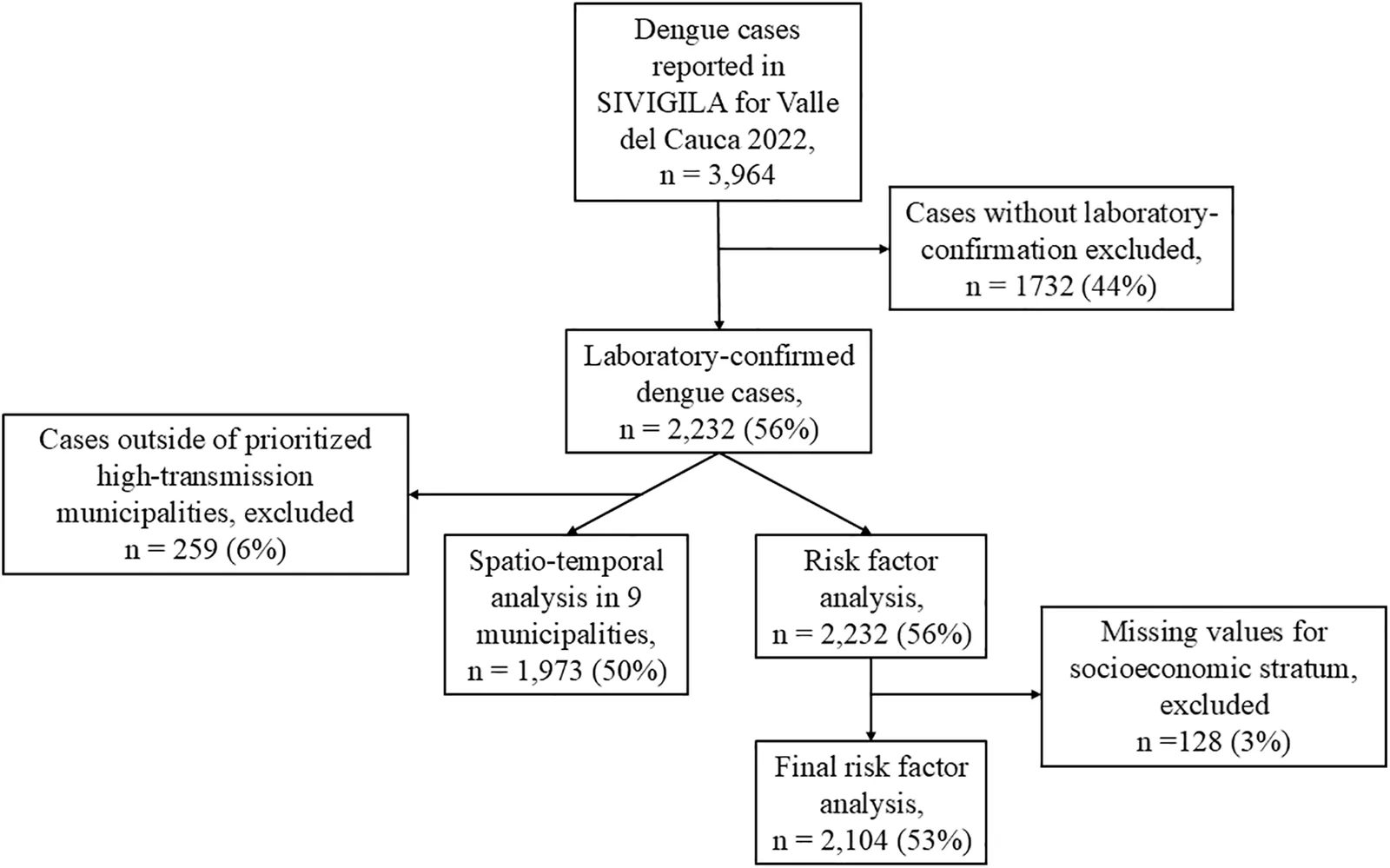
STROBE Flow Chart of included dengue cases. SIVIGILA, Sistema Nacional de Vigilancia en Salud Pública.

Among the nine municipal study areas considered, 28 statistically significant dengue clusters were detected in six (Table 1). Of these, 23 clusters were identified in Cali, and 1 cluster each in Palmira, Yumbo, Jamundí, Florida, and Tuluá. Candelaria had no eligible laboratory-confirmed cases in its defined study area and was not scanned with SaTScan. No significant clusters were detected in Buga or Cartago. Cluster radii ranged from 0 to 0.98 km. Across all study areas, 632 of 1,973 dengue cases (32%) fell within clusters while the other dengue cases occurred dispersed outside of clusters.

**Table 1:** Dengue clusters detected in municipalities from Valle del Cauca, Colombia in 2022.

Municipality	Time frame	Total clusters	Cases in study area, n (% within clusters)	Median cluster population, n (IQR)	Median cluster radius, km (IQR)	Median cases in cluster, n (IQR)	Median relative risk (IQR)
Cali	Jan-Dec	23	1,771 (32.0)	3,412 (716–6,717)	0.29 (0.11–0.47)	15 (12.5–23.5)	11.86 (6.45–46.58)
Palmira	Jun-Nov	1	45 (22.2)	2,558	0.62	10	9.52
Yumbo	Jun-Nov	1	41 (61.0)	6,659	0.95	25	14.29
Jamundí	Jan-May	1	36 (27.8)	478	0.13	10	45.79
Buga	No cluster	0	36 (0.0)	NA	NA	NA	NA
Tuluá	Jan-Jun	1	18 (55.6)	45	0.00	10	1,561.04
Florida	Jul-Dec	1	16 (62.5)	1,002	0.29	10	27.46
Cartago	No cluster	0	10 (0.0)	NA	NA	NA	NA
Candelaria	No scan	NA	0 (NA)	NA	NA	NA	NA

Significance level p < 0.05; 9,999 Monte Carlo replications. Cluster sizes ≥ 10 cases. Maximal spatial window: 20% of the study population at risk and maximal cluster size 1 km. Maximal temporal window: 50% of study period. Time frame uses 1-month units. Relative risk indicates the likelihood that cases occur within the detected cluster compared to the remainder of the study area and study period. Candelaria had no eligible laboratory-confirmed cases within the study area and was not scanned. Single-cell clusters have a SaTScan radius of zero. The population raster resolution per cell was approximately 100 × 100 m. IQR, interquartile range; RR, relative risk; NA, not applicable.

Using a monthly time scale, clusters were detected between January and December 2022. The 28 significant clusters lasted 1 month (n = 1), 2 months (n = 1), 3 months (n = 3), 4 months (n = 1), 5 months (n = 2) and 6 months (n = 20). Twenty clusters (71.4%) reached the maximum of 6 months. Dengue clusters in Cali occurred throughout the year (Fig 3). The single clusters outside Cali occurred in January–May in Jamundí, January–June in Tuluá, June–November in Palmira and Yumbo, and July–December in Florida. Of the 28 dengue clusters identified, 1 (3.6%) was confined to the warm–dry seasons (December–February, June–September), 1 (3.6%) occurred only within the cool–wet season (March–May, October–November), and 26 (92.9%) spanned both climatic seasons (Fig 4). During a sensitivity analysis with maximum windows of 3 and 1 months, 18 and 13 significant clusters were detected, containing 444 (22.5%) and 190 (9.6%) of the 1,973 cases, respectively, compared with 632 (32.0%) in the primary analysis. Median cluster relative risks were 14.97, 8.99, and 18.60 for the 6-, 3-, and 1-month maxima. Thus, localized clustering remained evident, but its extent and specific cluster definitions depended on the maximum temporal window (S3 Table).

**Fig 3:**
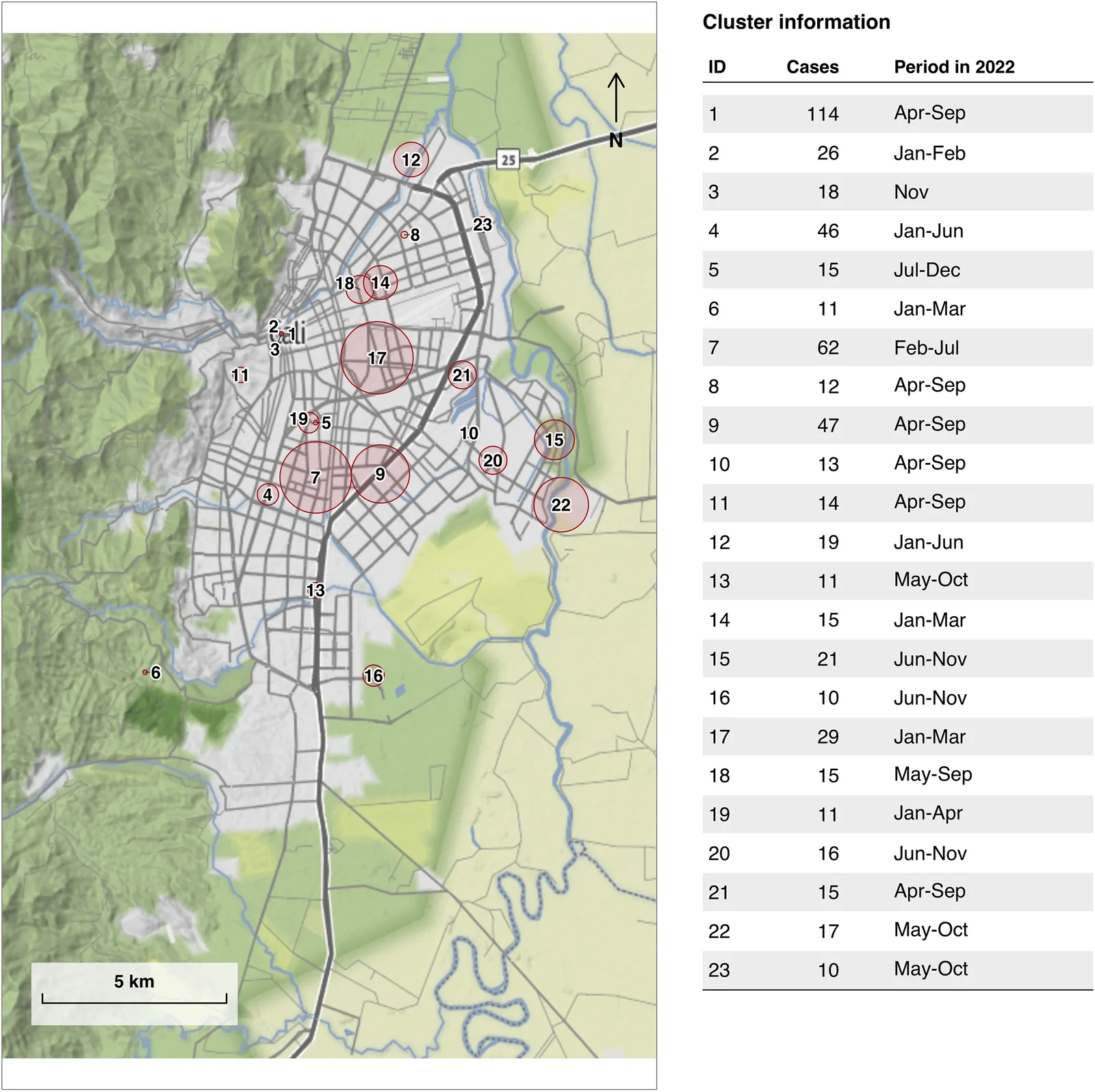
Statistically significant spatio-temporal dengue clusters in Cali, Colombia, 2022. Map showing statistically significant dengue clusters detected in Cali. Maximal spatial window: 20% of study population at risk. Maximal temporal window: 50% of study period. Red circles indicate dengue clusters and their radii. Labels 1–23 retain the SaTScan cluster identifiers. The table shows the number of observed cases and the months in which each cluster occurred. All clusters were significant at p < 0.05 using 9,999 Monte Carlo replications. The Cali municipal boundary was obtained from the GADM Database of Global Administrative Areas, version 4.1, Colombia administrative level 2. Direct boundary dataset: https://geodata.ucdavis.edu/gadm/gadm4.1/shp/gadm41_COL_shp.zip. Basemap tiles Stadia Maps; Stamen Design (CC BY 4.0), OpenMapTiles; OpenStreetMap contributors (ODbL). See stadiamaps.com/attribution, openmaptiles.org, openstreetmap.org.

**Fig 4:**
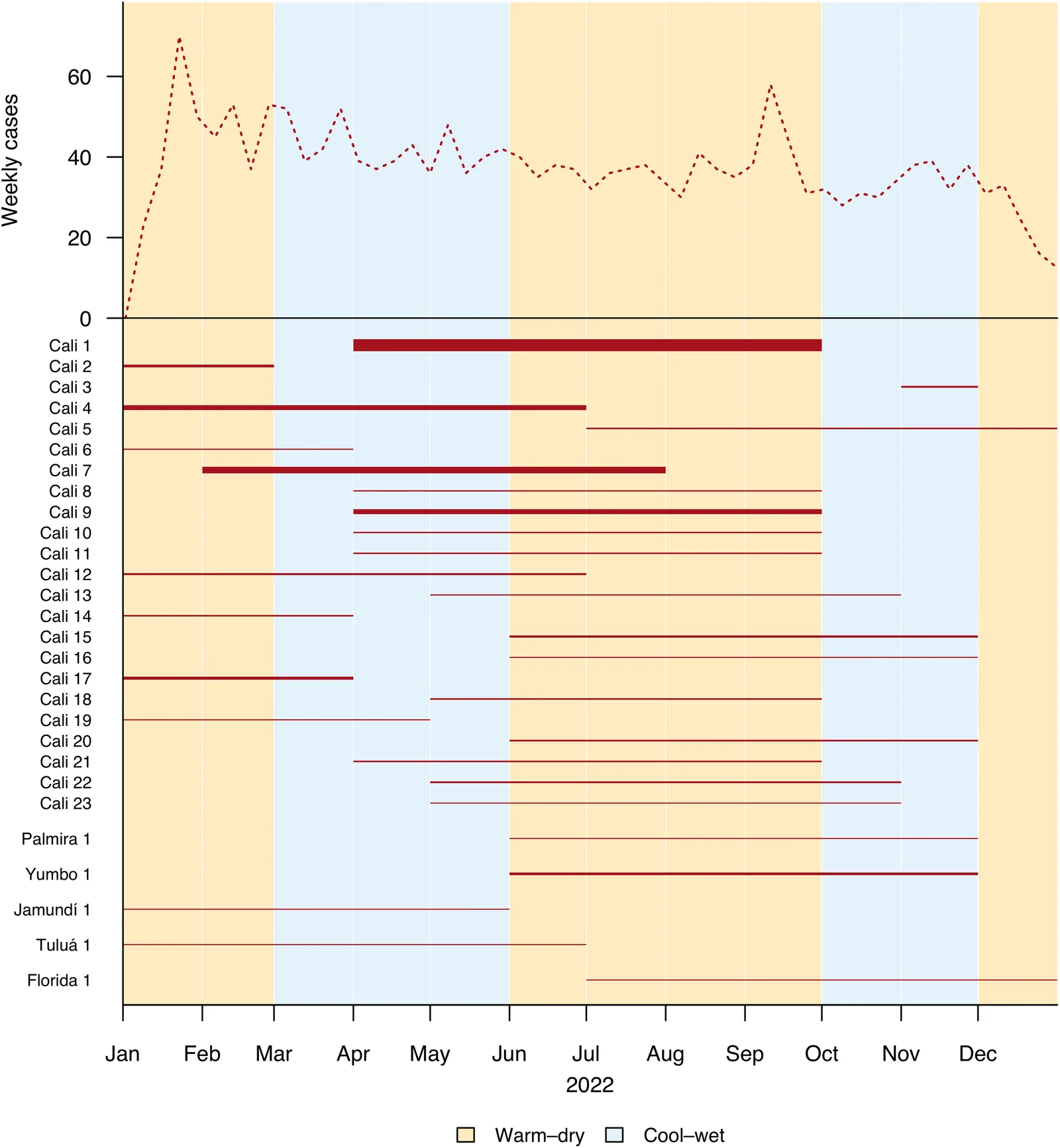
Weekly dengue cases and dengue clusters in Valle del Cauca, Colombia, 2022. Weekly dengue cases (red dotted line) and dengue cluster durations (red bars, thickness proportionate to cases in cluster) by municipality in Valle del Cauca, Colombia, January-December 2022. The black horizontal line separates the epidemic curve (above) from clusters (below). Clusters are grouped by municipality and labeled with their respective SaTScan identifiers. Orange shading marks warm-dry seasons (Dec–Feb, Jun–Sep) and blue shading marks cool-wet seasons (Mar–May, Oct–Nov).

### Risk factors for hospitalization among dengue patients

The demographic and clinical characteristics of laboratory-confirmed dengue cases, stratified by the variable of interest SES, are presented in Table 2. Of the 2,104 cases included in the analysis, 1,248 (59%) belonged to the low SES (SES 1–2) group, while 856 (41%) were classified as medium-high SES (SES 3–6).

**Table 2:** Demographic and Clinical Characteristics by Socioeconomic Stratum Group Among Laboratory-Confirmed Cases in Valle del Cauca, Colombia, N = 2,104^1.^

Characteristic	Low SES (1–2) N = 1248	Medium-High SES (3–6) N = 856	P-value
**Age (years)**			**<0.001**
Median (IQR)	14 (7-31)	19 (10-39)	
**Sex**			**0.321**
Male	666 (53.4%)	438 (51.2%)	
Female	582 (46.6%)	418 (48.8%)	
**Ethnicity**			**<0.001**
Black/Afro-Colombian	46 (3.7%)	6 (0.7%)	
Other^2^	1,202 (96.3%)	850 (99.3%)	
**Severity of Disease**			**0.009**
Without warning signs	704 (56.4%)	535 (62.5%)	
With warning signs	515 (41.3%)	310 (36.2%)	
Severe dengue	29 (2.3%)	11 (1.3%)	
**Hospitalization**			**<0.001**
Yes	627 (50.2%)	333 (38.9%)	
No	621 (49.8%)	523 (61.1%)	
**Clinical Care Setting**			**<0.001**
Ambulatory	537 (43.0%)	503 (58.8%)	
General floor	480 (38.5%)	258 (30.1%)	
ICU	59 (4.7%)	34 (4.0%)	
Observation Unit	116 (9.3%)	46 (5.4%)	
Referral to hospital	56 (4.5%)	15 (1.8%)	
**Insurance Status**			**<0.001**
No insurance	20 (1.6%)	14 (1.6%)	
Contributory	711 (57.0%)	739 (86.3%)	
Subsidized	498 (39.9%)	70 (8.2%)	
Other	19 (1.5%)	33 (3.9%)	

Demographic and clinical characteristics of patients by socioeconomic stratum group among laboratory-confirmed dengue cases in Valle del Cauca, Colombia, 2022. Bivariate analyses: Wilcoxon-Rank for age. Fisher exact test for ethnicity. All others: Chi-square test. IQR, interquartile range; ICU, intensive care unit.

^1^Missing values stratum: n=128 (5.7% of laboratory-confirmed cases).

^2^Other, Indigenous, ROM/Gitano, Raizal, Palenquero.

The median age of cases in the low SES group was 14 years (IQR: 7–31), significantly lower than the median age of 19 years (IQR: 10–39) in the medium-high SES group (p < 0.001). There was no significant difference in sex distribution between the two groups (p = 0.321). However, ethnicity differed significantly by SES, with a higher proportion of Black/Afro-Colombian individuals in the low SES group (3.7%) compared to the medium-high SES group (0.7%) (p < 0.001). Disease severity and hospitalizations also varied between the groups. A greater proportion of dengue cases in the low SES group presented with warning signs (41.3% vs. 36.2%) and hospitalization was significantly more frequent among individuals in the low SES group (50.2% vs. 38.9%, p < 0.001).

Multivariable logistic regression was used to examine the association between clinical and sociodemographic characteristics and hospitalization among laboratory-confirmed dengue cases in Valle del Cauca (Table 3). Model variables were included due to reaching a significance level of p ≤ 0.25 in the bivariate analyses or by clinical relevance (sex).

**Table 3:** Association of Sociodemographic Characteristics with Hospitalization among Laboratory-Confirmed Dengue Cases in Valle del Cauca, Colombia, N = 2,104^1.^

Characteristic	OR [95% CI]	P-value
Low Stratum (1–2)	1.30 [1.07 – 1.57]	0.009
**Sex**		
Female	1.12 [0.94 - 1.34]	0.21
**Ethnicity**		
Black/Afro-Colombian	1.46 [0.82 - 2.61]	0.20
**Insurance Status**		
No insurance	3.41 [1.61 – 7.23]	0.001
Subsidized	1.74 [1.41 – 2.15]	<0.001
Other	0.66 [0.36 – 1.21]	0.18
**Age**		
< 10	1.07 [0.79 – 1.45]	0.64
10–19	1.18 [0.87 – 1.61]	0.28
30–39	0.75 [0.50 – 1.11]	0.15
40–49	0.56 [0.36 – 0.89]	0.013
50–59	0.77 [0.49 – 1.21]	0.26
60–69	1.15 [0.72 – 1.85]	0.55
≥ 70	2.47 [1.51 – 4.04]	< 0.001

Association of sociodemographic characteristics with hospitalization among laboratory-confirmed dengue cases in Valle del Cauca, Colombia, 2022. Primary multivariable logistic regression model without adjustment for severity. Hosmer-Lemeshow p = 0.94. Link-test squared term p = 0.41. Reference categories: socioeconomic strata 3–6; male sex; other ethnicity (Other, Indigenous, ROM/Gitano, Raizal, and Palenquero); contributory insurance; and age 20–29 years. OR, Odds Ratio; CI, Confidence Interval.

^1^Missing values stratum: n=128 (5.7% of laboratory-confirmed cases).

In the final multivariable logistic regression model, low SES was significantly associated with hospitalization, with individuals in this group having 30% higher odds of being hospitalized (OR: 1.30, 95% CI: 1.07–1.57, p = 0.009). Being uninsured or being enrolled in the state-subsidized insurance scheme was also significantly associated with hospitalization (OR: 3.41, 95% CI: 1.61–7.23, p = 0.001 and OR: 1.74, 95% CI: 1.41–2.15, p < 0.001, respectively). Compared to the reference age group (20–29 years), individuals aged 70 and above had higher odds of hospitalization (OR: 2.47, 95% CI: 1.51–4.04, p < 0.001). Among laboratory-confirmed cases, 112 (5.0%) were aged ≥70 years. No significant associations were observed for sex and being Black/Afro-Colombian.

Clinical care setting was not included in the model because it overlaps conceptually with hospitalization. Insurance was identified as a potential confounder for the association of socioeconomic stratum with hospitalization. An exploratory SES-by-severity interaction could not be fully estimated because all 11 patients with severe dengue in strata 3–6 were hospitalized, causing separation. Table 3 presents the sociodemographic model without adjustment for severity. For this model, the Hosmer–Lemeshow test did not detect lack of fit (p = 0.94) and the link-test squared term was not significant (p = 0.41). Additional adjustment for dengue severity showed an OR for low SES of 1.57 (95% CI 1.17–2.11), compared with 1.30 (95% CI 1.07–1.57) without severity. The severity-adjusted model showed lack of fit on the Hosmer–Lemeshow test (p = 0.0045) and a significant link-test squared term (p = 0.002). Severity was not included in the primary model because it is closely related to the admission decision.

In a sensitivity analysis that expanded the case definition to include all notified dengue cases irrespective of laboratory confirmation (S1 Table), the association between low SES (strata 1–2) and hospitalization was attenuated and its confidence interval included 1 (OR 1.11, 95% CI 0.95–1.30), whereas the higher odds of hospitalization among uninsured and subsidized patients remained. The association between age and hospitalization showed a more pronounced U-shaped pattern. Compared with adults aged 20–29 years, children and adolescents (<10 and 10–19 years) and older adults (60–69 and ≥70 years) had higher odds of hospitalization. In addition, the bivariate analysis showed that laboratory-confirmed cases differed from non–laboratory-confirmed cases in SES and clinical severity (S2 Table). Laboratory-confirmed cases were less likely to be from the lowest SES (strata 1–2: 59.3% vs 73.4%) and more likely to be from strata 3–6 (40.7% vs 26.6%). Laboratory-confirmed cases more often presented with warning signs (39.3% vs 29.3%) or severe dengue (1.9% vs 0.4%) and were hospitalized more frequently (46.1% vs 20.3%, overall hospitalization across all cases: 34.8%).

## Discussion

This study considered nine municipal study areas in Valle del Cauca, Colombia, in 2022 and identified spatio-temporal clustering of laboratory-confirmed dengue cases at the sub-municipal level in six. The 28 statistically significant clusters with radii ranging from 0 to 0.98 km lasted from one to six months, and contained 32.0% of spatially analyzed laboratory-confirmed cases. In some municipalities, a high proportion of analyzed dengue cases occurred within a small number of clusters, indicating that transmission is focal in space and time rather than evenly distributed across neighborhoods. Lower socioeconomic stratum (SES 1–2), being uninsured or enrolled in the state-subsidized insurance scheme, and age ≥ 70 years were associated with higher odds of hospitalization for dengue. These findings highlight the potential of spatio-temporal mapping using the Kulldorff method at the sub-municipal level to support public health officials in prioritizing limited resources for optimizing vector control measures and underscore potential sociodemographic disparities in dengue hospitalization in Colombia. Other statistical approaches have been applied in the context of dengue. Bayesian space-time conditional autoregressive (CAR) models smooth risk across neighboring areal units and are useful for estimating relative risk surfaces [17], but they require a pre-specified neighborhood structure and produce continuous risk maps that are harder to convert into discrete intervention zones. Elliptical or irregularly shaped scan statistics, such as FleXScan, can identify non-circular clusters [32]. However, this added flexibility can increase computational demands and produce irregular cluster boundaries that are less straightforward to translate into field operations. By contrast, Kulldorff’s circular space-time scan statistic produces compact clusters with defined start and end dates, making the results easier to communicate to the Secretaría de Salud and to translate into reactive vector-control activities. The selected spatial and temporal window limits influence the size and location of detected clusters, and the 1 km radius cap was a deliberate operational constraint. Future studies using complementary statistical approaches could help confirm persistent high-risk neighborhoods.

Clusters were detected throughout the year. Eastin et al. previously observed higher dengue transmission during the warm-dry periods in Cali [23]. In this analysis, 26 of 28 clusters (92.9%) spanned both warm–dry and cool–wet seasons, but 20 (71.4%) reached the six-month maximum. Crossing seasons is partly determined by the permitted temporal maximum duration and is not independent evidence of sustained transmission. However, local conditions may sustain elevated risk across seasonal transitions. Potential contributors include household water storage, deficient solid-waste management and water-retaining street catch basins [33], dense housing and other socio-environmental conditions in low-stratum neighborhoods [14,22], and daily mobility between home, work, and school. Surveillance and analytical factors could also play a role, since the 50% temporal upper bound allowed clusters to extend across more than one seasonal period and improved ascertainment during outbreaks could lengthen apparent cluster duration. Therefore, these multi-month clusters identify residential case concentrations. Their duration does not establish uninterrupted transmission and may reflect surveillance processes and the selected scan-statistic parameters. Prior spatial modeling in Cali showed that dengue risk varies across neighborhoods, supporting the plausibility of locally sustained transmission [17].

Cali accounted for 23 of the 28 detected clusters in our analysis, which corresponds with its larger population but also indicates localized transmission hotspots. These may be driven by population density [14], inadequate vector control measures, and environmental conditions that favor *Aedes* breeding [33]. Proximity to tire shops and plant nurseries has previously been identified as local drivers of dengue risk in Cali [14]. Because we specified a maximum cluster radius of 1 km, the spatial extent of clusters partly reflects this constraint. However, the concentration of reported dengue cases in sub-kilometer areas over extended periods may have operational implications, as the observed cluster sizes and durations could help define smaller catchment areas for intensified vector-control activities. The scan durations, most of which reached the six-month maximum, do not establish how long interventions should be sustained.

Our findings suggest that among patients with dengue in Valle del Cauca, lower socioeconomic status, lack of insurance, and enrollment in the state-subsidized insurance scheme were associated with higher odds of hospitalization. These associations may reflect differences in healthcare access, healthcare-seeking behavior or hospital admission practices which should be studied further. A prior study proposed that higher socioeconomic strata in Cali were associated with lower levels of dengue infection rates, but hospitalization rates were not evaluated [14]. For Cali, Carabali et al. found that state-subsidized health insurance and low SES were associated with higher dengue rates and severity [31]. Patients in low socioeconomic strata in Colombia are more likely to qualify for the state-subsidized health insurance scheme and may exhibit different hospitalization patterns due to disparities in healthcare access and utilization compared to those in medium or high socioeconomic strata with other types of health insurance schemes. Our findings suggest socioeconomic disparities in dengue management in Valle del Cauca. Additionally, age ≥ 70 years was associated with higher odds of hospitalization. The association between age ≥ 70 years and hospitalization is consistent with prior studies showing worse dengue outcomes with advancing age. A multinational cohort in Mexico, Brazil, and Colombia reported higher in-hospital mortality among hospitalized dengue patients with older age and comorbidities [34]. A systematic review identified diabetes mellitus and several clinical markers as risk factors for dengue mortality [35]. Population-based surveillance data from Taiwan during the 2014–2015 epidemic also showed progressively increasing risks of dengue-related hospitalization, intensive care unit admission, and death with advancing age [36]. Possible explanations include age-related immune changes, a higher burden of comorbidities, and cytokine-mediated inflammation and vascular leakage in severe dengue [37,38]. In addition, PAHO clinical algorithms recommend closer monitoring and individualized management for older adults and patients with comorbidities [39]. Our finding supports close outpatient follow-up and early reassessment of older adults with dengue.

Our sensitivity analyses indicate that the association between socioeconomic stratum and hospitalization depends on the dengue case definition. When we included all notified cases irrespective of laboratory confirmation, the association with low SES was attenuated, while the higher odds among uninsured patients and those with state-subsidized insurance schemes persisted. Bivariate analyses showed that laboratory-confirmed cases were more likely to present with warning signs or severe dengue and were more frequently hospitalized than non–laboratory-confirmed cases, suggesting that laboratory testing may be performed more often for higher-acuity clinical presentations and in hospital care settings. In contrast, non–laboratory-confirmed notifications may capture a broader clinical spectrum during high-transmission periods, including milder dengue and possibly non-dengue febrile illnesses classified as dengue, which could attenuate the observed associations toward the null. The more pronounced U-shaped age pattern in the expanded case definition could reflect clinical decision-making, as children and older adults may be admitted more readily because of perceived vulnerability. Overall, these findings suggest that risk factors for hospitalization for dengue are sensitive to case definition and may reflect a combination of differences in severity, diagnostic ascertainment, and care-seeking or admission practices.

This study has several limitations. For the spatial analysis, the use of circular scan clusters may have resulted in the inclusion of low-risk areas within high-risk clusters, because the circular shape may not precisely match the true geographic distribution of risk, which can be irregular. In addition, the population denominator was derived from the WorldPop 100 m raster, which is modeled rather than directly enumerated and may differ locally from DANE census projections. This could bias relative risk estimates if local denominators were under- or overestimated. The 1 km maximum radius, 20% spatial window, 50% temporal window, and monthly aggregation were selected for operational reasons. The temporal sensitivity analysis showed that shorter maximum windows reduced the proportion of cases included in significant clusters, from 32.0% with a 6-month maximum to 22.5% and 9.6% with 3- and 1-month maxima. Twenty of 28 primary clusters reached the 6-month maximum, limiting inference about when elevated risk ended. The 6-month maximum was retained for the retrospective identification of elevated dengue risk during the calendar year. The results do not establish it as an optimal window for outbreak alerts. Smaller spatial caps, irregular-shape scans, or finer temporal aggregation could also yield different clusters. Clustering over multiple months should not be interpreted as evidence of a single uninterrupted transmission chain. The study also relied on passive surveillance data from SIVIGILA, which may underestimate the true burden of dengue because of underreporting or misdiagnosis, especially during co-circulation with chikungunya and Zika [9,18]. Restricting the primary analysis to laboratory-confirmed cases aimed to reduce clinical misclassification but may have introduced diagnostic-ascertainment bias if testing was more likely among patients with warning signs, severe disease, or health-facility admission. Missing data for the included variables were less than 10%, but bias due to missing data and possible reporting errors to SIVIGILA remains possible. In addition, socioeconomic stratum 3 was included in the comparator group because strata 4–6 were sparse. Because stratum 3 is classified as “medium-low” and may share some subsidy-related characteristics with lower strata, this grouping may have attenuated the estimated association between low socioeconomic stratum and hospitalization. Furthermore, using patients’ residential addresses as a proxy for dengue infection site may not capture the true site of infection. Residential clusters should therefore be interpreted as clusters of reported case residence rather than definitive transmission foci. Dengue transmission patterns are complex, and this study did not account for human mobility, behavioral factors, or other socio-environmental determinants that may influence disease spread.

## Conclusions

Our study identified spatio-temporal clustering of dengue below the municipality level in Valle del Cauca, Colombia, with 28 clusters comprising 32% of spatially analyzed laboratory-confirmed cases, having radii of up to 1 km, lasting one to six months. These findings demonstrate the potential of geospatial analysis to inform reactive vector control interventions in collaboration with health officials. The association between lower SES and higher odds of hospitalization may indicate health disparities in dengue outcomes in Colombia. Our findings emphasize the need for equitable healthcare access and intervention strategies tailored to high-risk populations, including the elderly. Future research that integrates climate, entomological, and public health intervention data and examines whether clusters repeatedly appear in the same neighborhoods across multiple years could further refine spatio-temporal risk assessment and help distinguish areas with transient outbreaks from those with persistent elevated risk for dengue infection that may warrant intensified vector control or structural interventions in endemic regions.

## Supporting information

S1 Table Association of Sociodemographic Characteristics with Hospitalization among All Dengue Cases Irrespective of Laboratory Confirmation in Valle del Cauca, Colombia, 2022, N = 3579^1.^ Multivariable logistic regression model showing the association between sociodemographic risk factors and hospitalization among all notified dengue cases in Valle del Cauca in 2022. Hosmer-Lemeshow p = 0.40. Link-test squared term p = 0.15. OR, Odds Ratio; CI, Confidence Interval. Reference categories: socioeconomic strata 3–6; male sex; other ethnicity (Other, Indigenous, ROM/Gitano, Raizal, and Palenquero); contributory insurance; and age 20–29 years. ^1^Missing values stratum: n = 385 (9.7% of total cases).
(DOCX)

S2 Table Demographic and Clinical Characteristics of Patients with Dengue by Laboratory Confirmation Status in Valle del Cauca, Colombia, 2022, N = 3964. Demographic and clinical characteristics of patients by laboratory confirmation group among dengue cases in Valle del Cauca, Colombia, 2022. Bivariate analyses: Wilcoxon-Rank for age. Fisher exact test for ethnicity. All others: Chi-square test. ^1^Missing values stratum: n=385 (9.7%). ^2^Other, Indigenous, ROM/Gitano, Raizal, Palenquero.
(DOCX)

S3 Table Sensitivity of the dengue space-time scan to maximum temporal windows of 1, 3, and 6 months in Valle del Cauca, Colombia, 2022. Scans used 1,973 cases, 20% maximum population window, 1 km maximum radius, minimum 10 cases, monthly time units, 9,999 Monte Carlo replications, and p < 0.05. Six-month scan represents the primary analysis. Warm–dry months: December–February and June–September, cool–wet months: March–May and October–November. Candelaria had no eligible laboratory-confirmed cases within the study area and was not scanned. Single-cell clusters have a SaTScan radius of zero. The population raster resolution is approximately 100 × 100 m. RR, relative risk; NA, not applicable.
(DOCX)
