# Supplementary material for: Spatio-temporal clustering of dengue in Valle del Cauca, Colombia
Source: PLoS Negl Trop Dis. 2026 Nov 10;20(11):e0014855. doi: 10.1371/journal.pntd.0014855 (PMC13766613; doi:10.1371/journal.pntd.0014855)
Supplement: Supplementary file 1 [file pntd.0014855.s004.pdf]

Reviewer

The authors present a spatio-temporal analysis of dengue cases to identify potential clusters that could help guide future health interventions in Valle del Cauca, Colombia. In addition, the study includes a multivariable regression analysis to identify factors associated with hospitalization. Overall, the study is clear, well structured, and easy to follow. However, several methodological and reporting aspects require more precision and justification to strengthen the robustness of the findings and their interpretation.

Comments by section

Introduction

1. Expand the epidemiological context of dengue in Colombia.

It would be useful to further expand Colombia's epidemiological profile (e.g., recent burden, trends, territorial heterogeneity) to highlight the public health situation in Colombia. For example, national surveillance reports describes the event's behavior and its indicators (incidence, confirmation, hospitalization), etc. Moreover, PAHO situation updates contextualize Colombia's dengue burden within the Americas region.

2. Expand the context on prevention and control in Colombia.

It is recommended to briefly describe how vector control actions, surveillance, and outbreak response are organized, and why a spatio-temporal clustering approach may provide operational value.

Methods

3. Justification of the "spatial window" (20% of the population) and the maximum radius (1 km). Line 202

It was mentioned that the selection was based on "discussions with stakeholders", which is valid as a programmatic criterion, but it should be complemented with evidence or references (or at least a more explicit technical justification), because these parameters strongly influence the size and location of clusters.

4. Maximum temporal window (50% of the period = 6 months), line 202

Why was the time window set to 6 months?

5. Specify the date used for the temporal analysis (symptom onset date or notification date).

This is determinant for the temporal component of the cluster.

6. Statistical significance and Monte Carlo replications (999).

999 is acceptable, but if several clusters and comparisons are reported, 9999 usually provides more stable p-values. Please justify the choice.

Results

7. Socioeconomic stratum (SES):

Why was SES categorized and analyzed as low SES (SES 1–2) versus middle-high (SES 3–6)? If grouping is used (e.g., "low/middle/high"), it should be justified, especially because stratum 3 in Colombia may receive the same or similar subsidies as strata 1 and 2.

8. Figure 3 (map of Cali without clusters).

The left map (without clusters) seems redundant if a map of the study area is already presented in Figure 1. The figure could be simplified. I would suggest keeping only the right map showing the identified clusters.

9. Migrant population / external origin.

Given the migratory dynamics Colombia has experienced in recent years, it would be pertinent to clarify whether the variable "migrant/external origin" was available in the SIVIGILA database and why it was not included in the analyses. Migration has also been considered a factor associated with transmission risk of dengue, so it would be worth discussing.

Discussion

10. Compare Kulldorff/SaTScan with other approaches.

When the authors state that the method has potential (line 435), it would be valuable to briefly discuss its advantages and limitations compared with other alternatives (this helps interpret why Kulldorff was chosen and what biases it may introduce, e.g., circular/elliptical shape).

11. Statement about sustained transmission beyond climate/seasonality: expand evidence. Line 447

In this line, authors mention: “This suggests that factors beyond seasonal temperature and dryness contribute to sustained transmission.” Which factors are you referring to?

12. Age and hospitalization: place findings in the literature.

The study showed that age ≥70 years is associated with hospitalization; this should be compared with prior evidence (in dengue, the risk of complications often increases with age and comorbidities). The authors could expand with more supporting evidence.

Limitations (requires strengthening with other studies)

13. Underreporting/misclassification: support with evidence.

If underreporting or imperfect diagnosis is proposed, it would be advisable to cite regional/Colombian evidence. Are there studies documenting underreporting or misclassification of arboviruses in Colombia? Or other countries?

14. Human mobility and place of infection vs residence.

This is very relevant. Using residential addresses may not reflect the true place of infection (e.g., at work or school). I recommend expanding this limitation and, if possible, discussing implications for interpreting clusters and targeting control efforts.
