# Supplementary material for: Spatio-temporal clustering of dengue in Valle del Cauca, Colombia
Source: PLoS Negl Trop Dis. 2026 Nov 10;20(11):e0014855. doi: 10.1371/journal.pntd.0014855 (PMC13766613; doi:10.1371/journal.pntd.0014855)
Supplement: Supplementary file 2 [file pntd.0014855.s005.pdf]

Reviewer Report

Summary

This manuscript presents a spatio-temporal analysis of laboratory-confirmed dengue cases in Valle del Cauca, Colombia, using Kulldorff's space-time scan statistic to detect sub-municipal clusters and multivariable logistic regression to assess risk factors associated with hospitalization. The study identifies 35 statistically significant clusters (radius 0.1–1 km) and demonstrates associations between hospitalization and lower socioeconomic status, subsidized insurance enrollment, and advanced age.

Overall, the manuscript is well-structured, methodologically sound, and clearly motivated by operational public health relevance. The integration of spatial clustering with equity-focused hospitalization analysis is a notable strength. The collaboration with local health authorities and the explicit operational framing enhance translational value.

The study is a solid contribution to the spatial epidemiology literature. However, several areas would benefit from clarification and strengthening prior to publication.

Major Comments

1. Literature Context and Positioning

The manuscript would benefit from a more comprehensive engagement with prior spatio-temporal clustering and spatial modeling studies on dengue and other arboviruses in Colombia, particularly in Cali.

A substantial body of work has examined spatial heterogeneity, vulnerability, and transmission dynamics in this setting. For example, socioeconomic and environmental determinants of dengue in Cali have been modeled using spatial statistical approaches [3], and earlier work examined spatio-temporal patterns of dengue fever in Cali using fine-scale data [1]. Vulnerability modeling using both statistical and expert-based approaches has also been conducted [2].

In addition, more advanced spatial modeling frameworks have been applied in this context, including space–time conditional autoregressive (CAR) models to estimate neighborhood-level dengue risk [5]. Space–time cluster detection and co-occurrence analysis for chikungunya and dengue have also been investigated nationally [4].

Beyond purely epidemiological modeling, research in Cali has incorporated community perceptions, knowledge–attitudes–practices (KAP) surveys, and public health stakeholder engagement to better understand arboviral dynamics and intervention feasibility [6, 7, 8]. A systematic review examining socioeconomic drivers of Aedes-borne diseases further contextualizes how structural inequality influences vector ecology and transmission risk [9].

Given this existing literature, the manuscript would benefit from more explicitly positioning its contribution relative to these prior studies. While the present work focuses on sub-municipal scan-

statistic clusters with an operational radius constraint, similar themes of neighborhood-level heterogeneity, transmission persistence, and equity have already been explored using complementary methodological frameworks.

Expanding the literature review would strengthen the manuscript in two ways:

- It would situate the study more clearly within the established spatial epidemiology literature in Cali and Colombia.
- It would allow the authors to more sharply articulate what distinguishes their contribution from prior modeling and clustering approaches.

Importantly, this manuscript does appear to offer meaningful advances — particularly through:

- the explicit operational constraint on cluster size (maximum 1 km),
- stakeholder-informed parameter selection,
- restriction to laboratory-confirmed cases in the primary analysis,
- and integration of individual-level hospitalization risk modeling.

These strengths should be clearly framed relative to prior Colombian applications to avoid any perception of overstated novelty.

2. Clarification of Novel Contribution

The methodological framework (Kulldorff scan statistic with a Poisson model, Monte Carlo simulation, 20% spatial window, 50% temporal window) is well established in the spatial epidemiology literature.

Given the prior applications of CAR models, vulnerability modeling, multi-disease cluster detection, and neighborhood-level risk estimation in Cali, the manuscript would benefit from explicitly clarifying what conceptual or methodological advance it offers.

Is the primary innovation:

- the operational translation of cluster size into vector-control–feasible intervention units?
- the linkage between spatial clustering and hospitalization disparities?
- the identification of season-spanning persistence at sub-kilometer scale?
- or the formal integration of stakeholder-informed analytical constraints?

Clarifying this would strengthen the manuscript's conceptual contribution.

3. Interpretation of Season-Spanning Clusters

The finding that 63% of clusters spanned both warm-dry and cool-wet seasons is compelling. However, the discussion could further elaborate on whether this pattern reflects:

- sustained localized transmission dynamics,
- the influence of the chosen maximum temporal window (50% of study period),
- surveillance artifacts,
- or socio-environmental stability in high-risk neighborhoods.

A deeper interpretation here would enhance epidemiological insight.

4. Methodological Robustness

The operationally motivated restriction to a maximum cluster radius of 1 km is well justified. However, a brief discussion of how results might vary under alternative spatial window specifications would improve methodological transparency.

Similarly, clarification regarding alignment between raster-derived population denominators and official census estimates would strengthen reproducibility.

5. Case Definition and Diagnostic Bias

The sensitivity analysis including epidemiologically linked cases is a strong component of the manuscript. However, laboratory confirmation may be more likely among hospitalized or severe cases. A clearer discussion of potential diagnostic ascertainment bias — and how this might influence cluster detection or hospitalization modeling — would enhance rigor.

Overall Recommendation

This is a well-executed and policy-relevant contribution. With stronger engagement with the substantial prior spatial epidemiology literature in Cali and clearer articulation of its unique contribution, the manuscript will be well positioned for publication.

I recommend **minor to moderate revision**.

References

[1] Delmelle, E., et al. (2013). Spatio-temporal patterns of dengue fever in Cali, Colombia. *International Journal of Applied Geospatial Research*, 4(4), 58–75.

[2] Hagenlocher, M., Delmelle, E., Casas, I., & Kienberger, S. (2013). Assessing socioeconomic vulnerability to dengue fever in Cali, Colombia: statistical vs expert-based modeling. *International Journal of Health Geographics*, 12(1), 36.

[3] Delmelle, E., Hagenlocher, M., Kienberger, S., & Casas, I. (2016). A spatial model of socioeconomic and environmental determinants of dengue fever in Cali, Colombia. *Acta Tropica*, 164, 169–176.

[4] Desjardins, M. R., Whiteman, A., Casas, I., & Delmelle, E. (2018). Space-time clusters and co-occurrence of chikungunya and dengue fever in Colombia from 2015 to 2016. *Acta Tropica*, 185, 77–85.

[5] Desjardins, M. R., et al. (2020). Space–time conditional autoregressive modeling to estimate neighborhood-level risks for dengue fever in Cali, Colombia. *American Journal of Tropical Medicine and Hygiene*, 103(5), 2040.

[6] Desjardins, M. R., et al. (2020). Knowledge, attitudes, and practices regarding dengue, chikungunya, and Zika in Cali, Colombia. *Health & Place*, 63, 102339.

[7] Casas, I., Desjardins, M., & Delmelle, E. (2024). Knowledge, attitudes, and practices toward dengue fever in Cali, Colombia. *Geographical Review*, 114(1), 70–98.

[8] Desjardins, M. R., et al. (2025). Utilizing perceptions of public health officials in Cali, Colombia to support surveillance of co-circulating vector-borne diseases. *Social Science & Medicine*, 118890.

[9] Whiteman, A., et al. (2020). Do socioeconomic factors drive Aedes mosquito vectors and their arboviral diseases? A systematic review. *One Health*, 11, 100188.
