# Supplementary material for: Spatio-temporal clustering of dengue in Valle del Cauca, Colombia
Source: PLoS Negl Trop Dis. 2026 Nov 10;20(11):e0014855. doi: 10.1371/journal.pntd.0014855 (PMC13766613; doi:10.1371/journal.pntd.0014855)
Supplement: Supplementary file 3 [file pntd.0014855.s007.pdf]

Response to Reviewers

Manuscript: Spatio-temporal clustering of dengue in Valle del Cauca, Colombia

We thank the Editor and the reviewers for their thoughtful, constructive, and detailed feedback. Their comments have substantially improved the clarity and contextualization of our manuscript. Please find below our responses to each comment. Line numbers refer to the revised manuscript (titled "Manuscript").

Reviewer #1

Introduction

Comment 1.1: *Expand the epidemiological context of dengue in Colombia (recent burden, trends, territorial heterogeneity).*

Response: We revised the Introduction to add concise epidemiological context for Colombia while keeping the focus on the 2022 Valle del Cauca analysis. The revised text now states that dengue is endemic in Valle del Cauca and that recurrent epidemics require timely information on where transmission is occurring. We also added that national dengue incidence has fluctuated substantially, with epidemic cycles in 2010, 2013, 2016, 2019, and 2023, and that dengue risk is geographically heterogeneous according to the Instituto Nacional de Salud high-risk municipality classification. To address the reviewer's request for regional context, we also added a brief sentence based on PAHO noting the record dengue burden reported in the Americas in 2024. These changes start at line 101 and continue at line 115 of the revised Manuscript.

Comment 1.2: *Expand the context on prevention and control in Colombia and why a spatio-temporal clustering approach may provide operational value.*

Response: We agree and revised the Introduction to better explain why sub-municipal cluster detection can support dengue control. The revised text links geographic heterogeneity in dengue risk and the INS high-risk municipality classification to the need to identify local transmission hotspots. This change complements the existing text on SIVIGILA reporting, WHO vector-control recommendations, and coordination of control measures by the Secretaría de Salud del Valle del Cauca. These changes start at line 104 and continue at line 115 of the revised Manuscript.

Methods

Comment 1.3: *Justify the spatial window (20% of the population) and the maximum radius (1 km). (orig. line 202)*

Response: We revised the Spatio-temporal Analysis subsection to clarify the rationale for these parameters. The revised text states that the choice of spatial and temporal windows reflected methodological convention and operational constraints. It also clarifies that the 20% upper bound on the population at risk and the 1 km maximum cluster radius were selected to support an operational signal for reactive vector-control measures at the neighborhood level. The Limitations section now notes that alternative specifications could yield clusters with different size, location, or duration. This change starts at line 239 of the revised Manuscript, with the related Limitations text beginning at line 572.

Comment 1.4: *Why was the maximum temporal window set to 6 months (50% of the period)? (orig. line 202)*

Response: We clarified the temporal-window rationale in the Methods. The revised text states that the maximum temporal window of 50% of the study period, corresponding to 6 months, allowed clusters to

span more than one transmission peak within the calendar year. This change starts at line 241 of the revised Manuscript.

Comment 1.5: *Specify the date used for the temporal analysis (symptom onset vs notification date).*
Response: We clarified that the temporal component of each case was the date of notification to SIVIGILA and that cases were aggregated by month. This change starts at line 234 of the revised Manuscript.

Comment 1.6: *999 Monte Carlo replications is acceptable, but 9,999 usually provides more stable p-values. Please justify.*
Response: We repeated the SaTScan analyses using 9,999 Monte Carlo replications while preserving the same discrete Poisson scan settings. Table 1 and Figures 3 and 4 now reflect these results. The overall interpretation was unchanged, but the number of significant clusters increased from 35 to 43, driven by additional clusters in Cali, and the highest p-value among reported significant clusters was 0.0487. The updated cluster counts appear in the Results at line 332, Table 1 begins at line 344, and the Figure 3 and Figure 4 captions begin at lines 364 and 372 of the revised Manuscript.

Results

Comment 1.7: *Why was SES categorized as low (1–2) vs middle-high (3–6)? Stratum 3 may receive subsidies similar to 1–2.*
Response: We revised the Methods to explain the rationale for the SES grouping. The revised text states that Colombia's socioeconomic stratification is an administrative classification used mainly for differential billing of public utilities, that DANE classifies strata 1 and 2 as "low-low" and "low," and that stratum 3 is classified as "medium-low." We also note that stratum 3 may be eligible for public-utility subsidies under certain regulatory conditions. Because strata 4–6 were sparse in our dataset, strata 3–6 were combined to provide a stable comparator group. We also cite prior Colombian dengue research that defined strata 1–2 as low SES. This change starts at line 245 of the revised Manuscript. The related limitation about possible attenuation starts at line 594.

Comment 1.8: *Figure 3: the left map (without clusters) is redundant with Figure 1; keep only the cluster map.*
Response: We replaced Figure 3 with a single map of Cali showing the identified clusters and removed the redundant panel. The caption was updated accordingly. The revised Figure 3 caption begins at line 364 of the revised Manuscript.

Comment 1.9: *Clarify whether "migrant/external origin" was available in SIVIGILA and why it was not analyzed; migration may affect transmission risk.*
Response: We agree that migration is an important variable to consider for transmission risk. Migrant status was present in the SIVIGILA data structure but had substantial missingness and was therefore not included in the regression or cluster analysis. Rather than interpret an incomplete variable, we addressed this limitation more broadly by noting that the study relied on passive surveillance data, may be affected by missing data and reporting errors, and did not account for human mobility, behavioral factors, or other socio-environmental determinants that may influence dengue spread. This limitation is addressed starting at line 592 of the revised Manuscript.

Discussion

Comment 1.10: *Compare Kulldorff/SaTScan with other approaches (advantages/limitations, e.g., circular/elliptical shape). (orig. line 435)*

Response: We expanded the Discussion to compare Kulldorff's circular space-time scan statistic with Bayesian space-time CAR models and irregularly shaped scans such as FlexScan. The revised text notes that CAR models are useful for estimating relative-risk surfaces but are harder to convert into discrete intervention zones. It also notes that FlexScan can identify non-circular clusters but may produce irregular boundaries that are less straightforward to translate into field operations. We then explain why Kulldorff's compact clusters with defined start and end dates were suitable for communication with the Secretaría de Salud and for reactive vector-control planning. This change starts at line 472 of the revised Manuscript.

Comment 1.11: *Which factors beyond seasonal temperature and dryness contribute to sustained transmission? (orig. line 447)*
Response: We expanded the Discussion to specify plausible non-seasonal contributors to sustained transmission. These include household water storage, deficient solid-waste management and water-retaining street catch basins, dense housing and other socio-environmental conditions in low-stratum neighborhoods, and daily mobility between home, work, and school. We also note that surveillance and analytical factors could contribute to apparent cluster duration. This change starts at line 488 of the revised Manuscript, with the list of potential contributors starting at line 497.

Comment 1.12: *Compare the age ≥70 hospitalization finding with prior evidence on age, comorbidity, and severe dengue.*
Response: We expanded the Discussion to place the age ≥70 finding in the context of prior dengue literature. The revised text cites evidence from a multinational cohort in Mexico, Brazil, and Colombia, a systematic review and meta-analysis on dengue mortality, and population-based surveillance from Taiwan. We also added possible explanations, including age-related immune changes, comorbidity burden, cytokine-mediated inflammation, and vascular leakage in severe dengue, and we cite PAHO guidance on closer monitoring of older adults and patients with comorbidities. This change starts at line 532 of the revised Manuscript.

Limitations

Comment 1.13: *Support underreporting/misclassification with regional/Colombian evidence.*
Response: We expanded the Limitations to state that SIVIGILA is a passive surveillance system and may underestimate the true dengue burden because of underreporting or misdiagnosis, especially during co-circulation with chikungunya and Zika. We supported this point with Colombian evidence. We also clarified that restricting the primary analysis to laboratory-confirmed cases reduced clinical misclassification but may have introduced diagnostic-ascertainment bias if testing was more likely among patients with warning signs, severe disease, or health-facility admission. This change starts at line 585 of the revised Manuscript.

Comment 1.14: *Residential addresses may not reflect the true place of infection; expand this limitation and its implications.*

Response: We expanded the Limitations to clarify that residential addresses were used as a proxy for the probable site of infection and may not capture the true site of infection. The revised text states that residential clusters should be interpreted as clusters of reported case residence rather than definitive transmission foci. We also note that the study did not account for human mobility, behavioral factors, or other socio-environmental determinants that may influence disease spread. This change starts at line 598 of the revised Manuscript.

Reviewer #2

Major Comments

Comment 2.1: *Engage more comprehensively with prior spatio-temporal and spatial-modeling studies of dengue/arboviruses in Colombia and Cali, and position the contribution.*

Response: We expanded the Introduction to cite prior fine-scale spatio-temporal analyses in Cali, socioeconomic and environmental determinant models, space-time CAR models, national chikungunya and dengue co-occurrence analyses, KAP and stakeholder-engagement work, and a systematic review on socioeconomic drivers of Aedes-borne disease. We then clarified how the present study differs from this earlier work. This change starts at line 132 of the revised Manuscript.

Comment 2.2: *Clarify the novel conceptual or methodological contribution given prior applications in Cali.*

Response: We clarified that the contribution of the study is operational rather than methodological. The revised text states that the study applies an established scan-statistic method with a sub-kilometer radius, stakeholder-informed parameter selection, restriction to laboratory-confirmed cases, and patient-level hospitalization modeling. This change starts at line 144 of the revised Manuscript.

Comment 2.3: *Interpret the season-spanning clusters (sustained transmission vs temporal window vs surveillance vs socio-environmental stability).*

Response: We expanded the Discussion to address these interpretations. The revised text interprets multi-month persistence as compatible with locally sustained transmission while acknowledging that the 50% temporal upper bound, improved ascertainment during outbreaks, and socio-environmental conditions in high-risk neighborhoods could also contribute. The Conclusions also note that multi-year analyses could help distinguish transient outbreaks from areas with persistent elevated risk. This change starts at line 488 of the revised Manuscript, with the future-research statement beginning at line 611.

Comment 2.4: *Discuss sensitivity to alternative spatial windows and alignment of raster denominators with census estimates.*

Response: We revised the Limitations to note that the WorldPop 100 m raster is modeled and may differ locally from DANE census projections, which could bias relative-risk estimates if local denominators were under- or overestimated. We also added that the 1 km radius, 20% spatial window, 50% temporal window, and monthly aggregation were selected for operational reasons and that alternative specifications could yield clusters with different size, location, or duration. This change starts at line 572 of the revised Manuscript.

Comment 2.5: *Laboratory confirmation may be more likely among severe/hospitalized cases; discuss diagnostic-ascertainment bias.*

Response: We expanded the Limitations to state that restricting the primary analysis to laboratory-confirmed cases reduced clinical misclassification but may have introduced diagnostic-ascertainment bias if testing was more likely among patients with warning signs, severe disease, or health-facility admission. We performed a sensitivity analysis including all notified dengue cases, irrespective of laboratory confirmation status, to assess the robustness of the dengue case definition. This limitation starts at line 589 of the revised Manuscript, and the sensitivity-analysis results begin at line 445.

Reviewer #3

Methods

Comment 3.1: *Objectives are clear, but a formal testable hypothesis is not explicitly stated.*

Response: We added a formal hypothesis to the final paragraph of the Introduction. The revised text states that laboratory-confirmed dengue cases would show localized clustering in space and time at the sub-municipal level, and that dengue-related hospitalization would vary by socioeconomic stratum. This change starts at line 154 of the revised Manuscript.

Comment 3.2: *Design, population, sample size, statistical analyses, and ethics are appropriate.*

Response: Thank you. No content change was required.

Results

Comment 3.3: *Analysis matches the plan and results are clearly presented.*

Response: Thank you. No content change was required. We have re-checked that the Results are reported in the same order as the two stated aims and that all cluster counts now reflect the 9,999-replication analysis.

Comment 3.4: *Incomplete parenthesis in the hospitalization-by-SES sentence.*

Response: Corrected. The hospitalization-by-SES sentence now includes the closing parenthesis after the p-value. The corrected sentence appears at line 408 of the revised Manuscript.

Comment 3.5: *Improve final formatting and resolution of maps and tables.*

Response: Figures 3 and 4 were regenerated at 600 dpi, cluster labels were adjusted, and tables were reformatted. Figure 3 and Figure 4 captions begin at lines 364 and 372 of the revised Manuscript and figures are uploaded separately. Figures were optimized using the suggested PLOS NAAS tool.

Conclusions

Comment 3.6: *Conclusions, limitations, and public health relevance are consistent and not overstated.*

Response: Thank you. No change to the Conclusions was required. The Limitations were expanded in response to Reviewers 1 and 2, beginning at line 572 of the revised Manuscript. The updated Conclusions begin at line 600.

Editorial and Data-Presentation Comments

Comment 3.7: *Subject–verb agreement in the Introduction ("targeted reactive interventions … requires").*

Response: Corrected. "Requires" was changed to "require." The correction is at line 121 of the revised Manuscript.

Comment 3.8: *Typographical issue in the Figure 1 legend ("municipalitas").*

Response: Corrected to "municipalities." The correction is at line 176 of the revised Manuscript.

Comment 3.9: *Trailing comma at the end of the abstract.*

Response: Corrected. The final sentence of the Abstract now ends with a period. The correction is at line 71 of the revised Manuscript.

Comment 3.10: *Stray character before the paragraph introducing Table 3.*

Response: Corrected. The stray character was removed before the paragraph introducing Table 3. The corrected paragraph begins at line 377 of the revised Manuscript.

Comment 3.11: *Standardize decimal notation ("3,2%").*

Response: Corrected to "3.2%." The correction is in the Table 3 footnote at line 403 of the revised Manuscript.

Comment 3.12: *Standardize presentation of percentages in supplementary tables.*

Response: Corrected. Percentages in the supplementary tables were harmonized with the main-text tables. The Supporting Information captions begin at line 780 of the revised Manuscript, and the separate S1 and S2 files were updated accordingly.

Comment 3.13: *"Februay" typo in reference 1.*

Response: Corrected. The typo was corrected in the PAHO web-reference entry, now listed as Reference 3. Reference 3 begins at line 635 of the revised Manuscript.

Comment 3.14: *"Avaiable" typo in reference 21.*

Response: Corrected to "Available." Reference 21 begins at line 705 of the revised Manuscript.

Comment 3.15: *Standardize repeated punctuation in web references.*

Response: Corrected. The web references were reviewed for repeated punctuation, consistent "Accessed on:" phrasing, date formatting, and URL formatting. The reference list begins at line 625 of the revised Manuscript.

Comment 3.16: *Align the data-availability statement with journal policy language.*

Response: We revised the data-availability statement to align with journal policy language. The suggested revised statement specifies that the data are individual-level SIVIGILA surveillance records containing identifiable information, including residential addresses, and that the data are owned by the Secretaría de Salud del Valle del Cauca. The statement explains that the data cannot be shared publicly because this would compromise patient privacy under Colombian regulations and the approved study protocol. De-identified data may be requested from the Secretaría de Salud del Valle del Cauca, subject to a data-use agreement. The statement is communicated to the editors and not part of the main manuscript.

We thank the Reviewers and the Editor again for their constructive feedback, which has substantially strengthened the manuscript. We look forward to your response.

Kind regards,
Hendrik Sy
(on behalf of the co-authors)
