# Supplementary material for: Spatio-temporal clustering of dengue in Valle del Cauca, Colombia
Source: PLoS Negl Trop Dis. 2026 Nov 10;20(11):e0014855. doi: 10.1371/journal.pntd.0014855 (PMC13766613; doi:10.1371/journal.pntd.0014855)
Supplement: Supplementary file 4 [file pntd.0014855.s008.pdf]

Response to Reviewers

Manuscript: Spatio-temporal clustering of dengue in Valle del Cauca, Colombia

We thank the Editor and the reviewers for their constructive feedback on the revised manuscript. Please find below our responses to each comment.

During verification of spatial preprocessing, we found an error that assigned all cases in each raster cell to its latest case date. We corrected this by retaining individual dates and aggregating counts by cell and date before monthly scanning. This materially changed the spatial results: the previously reported 43 clusters containing 1,058 cases (54%) became 28 clusters containing 632 of 1,973 cases (32%), and the largest cluster decreased from 193 to 114 cases. The concentration toward the end of the year was partly an artifact of assigning earlier cases to later dates. The corrected clusters occur throughout the year. Buga no longer has a significant cluster. The spatial cohort is unchanged at 1,973. Both supplied analysis datasets contain 3,964 notifications, which is now reported consistently. Table 1, Figures 2–4, an added S3 Table and the interpretation have been aligned with the corrected analysis. We also corrected the description of the temporal analysis in the manuscript: consultation date was used instead of notification date.

Reviewer #1

Comment 1.1: The study clearly states two main objectives: to identify spatio-temporal clusters of laboratory-confirmed dengue cases at the sub-municipal level and to assess associations between sociodemographic and clinical characteristics and dengue-related hospitalization. The retrospective observational design using SIVIGILA surveillance data is appropriate, and the study population, eligibility criteria, and selected municipalities are well described. The sample size appears sufficient, particularly for the hospitalization analysis, as the final sample exceeded the required number estimated in the power calculation. The statistical methods, including Kulldorff's space-time scan statistic and multivariable logistic regression, are appropriate and supported by sensitivity analyses and model diagnostics.

Response: Thank you. No additional change was required.

Comment 1.2: The analyses presented in the Results section are consistent with the stated analysis plan. Overall, the results are clearly and adequately presented. The figures and tables are of sufficient quality and facilitate the interpretation of the main findings. The tables summarize the results of the analyses examining factors associated with hospitalization.

Response: Thank you. We have retained the Results structure, revised Figure 3 and Table 3, and checked Figure 4 in response to the specific comments below.

Comment 1.3: The conclusions are well supported by the data presented and are consistent with the main findings of the study. The identification of localized spatio-temporal dengue clusters may provide useful information for health authorities to better prioritize and target future surveillance and vector-control activities. The limitations of the analyses are clearly acknowledged and discussed. The authors also explain how these findings contribute to a better understanding of dengue transmission at the sub-municipal level. The study has clear public health relevance, particularly in low- and middle-income settings where resources are limited and more precise identification of high-risk areas could support a more efficient allocation of prevention and control resources.

Response: Thank you. We updated the results and explained how the maximum temporal window affects their interpretation. Results: lines 369–385. Temporal-window limitations: lines 615–626.

Comment 1.4: Overall, the manuscript is clearly written, well structured, and easy to follow. The authors address an important public health question and use an appropriate study design and analytical approach to identify spatio-temporal dengue clusters and explore factors associated with hospitalization. The study objectives are clearly defined and adequately addressed by the analyses presented. A particular strength of the study is the identification of localized high-risk areas that could help public health authorities better prioritize surveillance and design more targeted vector-control interventions.

Response: Thank you for this assessment.

Reviewer #2

Comment 2.1: Yes, all is sound. But I still do not believe that 50% is an ok parameter in Satscan for an infectious diseases. If it corresponds up to six month, that is too long.

Response: We agree that the temporal maximum requires justification for the study objective. Six months is an upper bound in this retrospective analysis, not the duration of an individual infection or a real-time alert interval. After correcting case-date assignment, we repeated the analyses with 3- and 1-month maxima, preserving the case cohort, spatial inputs, monthly aggregation, minimum of 10 cases, significance threshold, and 9,999 Monte Carlo replications. The primary 6-month analysis identified 28 significant clusters containing 632 of 1,973 cases (32.0%). The 3-month analysis identified 18 clusters containing 444 cases (22.5%), the 1-month analysis identified 13 clusters containing 190 cases (9.6%). Cases were counted once across the union of significant clusters. The 3- and 1-month analyses retained 343 (54.3%) and 158 (25.0%) of the cases included in primary clusters. Twenty of 28 primary clusters (71.4%) reached the six-month maximum. We retained this maximum for the retrospective objective while acknowledging that clusters and their duration depend on the temporal maximum. The results do not establish uninterrupted transmission, when elevated risk ended, or an optimal interval for outbreak alerts or interventions. The Methods, Results, Limitations, and S3 Table report this comparison. S3 Table now includes the proportions of clusters reaching each temporal maximum: 71.4%, 83.3% and 100.0% for the 6-, 3- and 1-month analyses, respectively. Methods: lines 237–248. Results: lines 369–385. Limitations: lines 615–626.

Comment 2.2: The map with the clusters needs work.
The figure is visually overcrowded, making it difficult for the reader to identify the main spatial patterns. The large number of crossing leader lines, overlapping proportional symbols, and repeated text labels substantially reduces readability. I recommend redesigning the figure to improve visual hierarchy by minimizing leader lines, simplifying the labeling strategy, reducing redundancy in the annotations, and considering alternative approaches (e.g., direct labeling, separate panels for temporal information, or a companion table). These changes would greatly improve the clarity and interpretability of the map.

Response: Figure 3 now shows the 23 corrected Cali clusters with their SaTScan identifiers and an adjacent table of case counts and periods.

Reviewer #3

Comment 3.1: Yes. The authors have adequately addressed this comment by adding an explicit and testable hypothesis aligned with both study aims. No further revision is required.

Response: We thank the reviewer.

Comment 3.2: The analysis presented generally matches the analysis plan and the Results are organized consistently with the two stated aims. The revised cluster counts appropriately reflect the analysis using

9,999 Monte Carlo replications, and the hospitalization analysis and sensitivity analyses are presented in a logical sequence.

Response: Thank you. We have retained this structure and addressed the specific reporting and presentation points below. Spatial results: lines 334–385. Hospitalization results: lines 412–498.

Comment 3.3: The Results are largely clear and complete. However, two minor reporting issues should be addressed before acceptance. First, in Table 3, the estimate for female sex is reported as OR 1.19 (95% CI 0.94–1.34), with p=0.21. The confidence interval and p-value do not appear internally consistent and should be verified against the original regression output.

Response: The original female-sex OR was transcribed incorrectly. Verified output gives OR 1.12 (95% CI 0.94–1.34), p=0.21. We also corrected the uninsured OR, other-insurance p-value and age 30–39 estimate against the original output. The Hosmer–Lemeshow test of the primary model has p=0.94. The primary model's link-test squared term has p=0.41. For the all-notified model, Hosmer–Lemeshow has p=0.40, and the link-test squared term has p=0.15 (S1 Table). These results are now stated in the manuscript. For Table S2 the ethnicity Fisher exact p-value is 0.055. Table 3. Model diagnostics: lines 475–480 and 833–836. S1 and S2 Tables.

Comment 3.4: Second, the statement that "a significant interaction was observed between hospitalization and dengue severity" is not statistically clear because hospitalization is the dependent variable. The authors should specify the actual interaction term evaluated or revise this sentence.

Response: We withdraw the earlier claim of a significant interaction. Hospitalization was the outcome. The exploratory interaction was SES by severity. All 11 severe-dengue patients in strata 3–6 were hospitalized, causing separation and preventing estimation of the full interaction. The severity main-effect model showed a low-SES OR of 1.57 (95% CI 1.17–2.11), versus 1.30 (1.07–1.57) without severity. The severity-adjusted model showed lack of fit. Severity was not included in the primary sociodemographic model because it is closely related to the admission decision. We now note in the Results section that the final model did not account for differences in disease severity. Lines 470–482.

Comment 3.5: Figure quality has improved, but Figure 3 remains visually crowded, with several overlapping labels and leader lines that reduce readability.

Response: Figure 3 uses the 23 corrected Cali identifiers with an adjacent table.

Comment 3.6: Figure 4 also appears partially truncated in the compiled reviewer PDF. The authors or production team should verify that the complete figure, axes, time scale, municipality labels, and annotations are clearly visible in the final files.
These are minor reporting and presentation issues and do not alter the overall interpretation or conclusions of the study.

Response: Figure 4 has been recreated using the corrected case dates and cluster intervals. It shows the weekly counts of all 1,973 spatially analyzed cases and the 28 significant clusters. We will check the generated submission proof before submission.

Comment 3.7: The conclusions are generally supported by the data presented. The authors appropriately state that the identified sub-municipal clusters may help inform reactive vector-control activities, and the main Conclusions use cautious language when interpreting the association between socioeconomic status and hospitalization.

The limitations are clearly and comprehensively described. The revised manuscript acknowledges the constraints of circular scan windows, the use of modeled WorldPop denominators, sensitivity to the selected spatial and temporal parameters, passive-surveillance underreporting, diagnostic-ascertainment

bias, missing data, the socioeconomic-stratum grouping, residential address as a proxy for infection location, and the absence of mobility and other socio-environmental variables.

The manuscript also explains how the findings may advance understanding of dengue transmission by showing that reported cases may cluster at a small sub-municipal scale and persist across seasonal periods. The potential operational value for prioritizing limited vector-control resources and identifying populations with higher odds of hospitalization is clearly discussed, and the public health relevance is adequately addressed.

Response: Thank you. We updated the spatial results and the temporal-window limitation, as described above. Spatial results: lines 346–385. Temporal-window limitations: lines 615–626.

Comment 3.8: Reference categories in Table 3: Please state explicitly the reference categories for socioeconomic stratum, sex, ethnicity, insurance status, and age, either within the table or in a footnote. At present, some reference groups are only implied.

Response: We added the reference categories to the Table 3 legend: socioeconomic strata 3–6, male sex, other ethnicity (Other, Indigenous, ROM/Gitano, Raizal, and Palenquero), contributory insurance, and age 20–29 years.

Comment 3.9: The sentence beginning "Our findings suggest that among patients with dengue…" should be revised for subject–verb agreement and clarity. For example: "Our findings suggest that lower socioeconomic status, lack of insurance, and enrollment in the state-subsidized insurance scheme were associated with higher odds of hospitalization."

Response: Corrected. The sentence now reads: "Our findings suggest that among patients with dengue in Valle del Cauca, lower socioeconomic status, lack of insurance, and enrollment in the state-subsidized insurance scheme were associated with higher odds of hospitalization." Lines 561–563.

Comment 3.10: The statement should specify that access to de-identified data is subject to institutional review and a data-use agreement, as indicated in the response letter. The authors should also clarify whether the analytical code can be made publicly available, since it would not contain individual-level identifiable information.

Response: We will specify in the Data Availability Statement that access is subject to institutional review and a data-use agreement. We provide the R and Stata analysis scripts in a code repository via the link below.

We propose the following Data Availability Statement: The data are individual-level SIVIGILA surveillance records containing identifiable information, including residential addresses, and are owned by the Secretaría de Salud del Valle del Cauca. They cannot be shared publicly because this would compromise patient privacy under the applicable Colombian regulations and the approved study protocol. Requests for de-identified data may be submitted to the Secretaría de Salud del Valle del Cauca (Dirección de Salud Pública), Gobernación del Valle del Cauca, Cali, Colombia (contactenos@valledelcauca.gov.co). Access is subject to institutional review and a data-use agreement. The R and Stata analysis scripts are provided in the code repository:
https://doi.org/10.5281/zenodo.23383328

Comment 3.11: This is a relevant and well-executed study addressing the spatial and temporal concentration of dengue cases at a scale that may be useful for local public health action. The manuscript combines two complementary components: detection of sub-municipal spatio-temporal clusters of laboratory-confirmed dengue cases and assessment of sociodemographic factors associated with hospitalization. This combination strengthens the applied value of the work.

The principal contribution is operational rather than methodological. The study does not introduce a new spatial-statistical method, but it applies an established space-time scan statistic using stakeholder-informed spatial constraints intended to generate intervention areas that are potentially manageable by local health authorities. The restriction to laboratory-confirmed cases, the sub-kilometer cluster scale, and the linkage with patient-level hospitalization analysis distinguish the study from some previous spatial analyses in Cali and Colombia. The manuscript is generally clearly written, appropriately referenced, and scientifically sound. The conclusions are mostly proportional to the evidence, and the public health relevance is well articulated. The authors have responded constructively to the previous reviews and have substantially strengthened the manuscript. The remaining concerns are limited to minor issues of statistical reporting, wording, figure readability, and editorial clarity, as detailed in the preceding sections. I therefore recommend Minor Revision

Response: We revised the temporal-window interpretation and corrected the reporting issues described above. Temporal-window limitations: lines 615–626. Corrected estimates and model reporting: Table 3 and lines 450–482.

We thank the Reviewers and the Editor again for their constructive feedback, which has strengthened the manuscript. We look forward to your response.

Kind regards,

Hendrik Sy

(on behalf of the co-authors)
