# Supplementary material for: Spatio-temporal clustering of dengue in Valle del Cauca, Colombia
Source: PLoS Negl Trop Dis. 2026 Nov 10;20(11):e0014855. doi: 10.1371/journal.pntd.0014855 (PMC13766613; doi:10.1371/journal.pntd.0014855)
Supplement: Supplementary file 5 [file pntd.0014855.s001.docx]

**S1 Table. Association of Sociodemographic Characteristics with Hospitalization among All Dengue Cases Irrespective of Laboratory Confirmation in Valle del Cauca, Colombia, 2022, N=3579^1^**

| **Characteristic** | **OR [95% CI]** | **P-value** |
| --- | --- | --- |
| Low SES (stratum 1-2) | 1.11 [0.95–1.30] | 0.202 |
| **Sex** |  |  |
| Female | 1.17 [1.02–1.35] | 0.026 |
| **Ethnicity** |  |  |
| Black/Afro-Colombian | 0.96 [0.65–1.43] | 0.850 |
| **Insurance Status** |  |  |
| No insurance | 2.50 [1.45–4.31] | 0.001 |
| Subsidized | 1.68 [1.43–1.98] | <0.001 |
| Other | 0.81 [0.48–1.38] | 0.445 |
| **Age (reference 20–29)** |  |  |
| < 10 | 1.44 [1.14–1.81] | 0.002 |
| 10–19 | 1.47 [1.16–1.87] | 0.001 |
| 30–39 | 0.70 [0.52–0.94] | 0.018 |
| 40–49 | 0.75 [0.53–1.06] | 0.108 |
| 50–59 | 1.00 [0.70–1.41] | 0.979 |
| 60–69 | 1.88 [1.29–2.74] | 0.001 |
| ≥ 70 | 3.48 [2.42–5.00] | <0.001 |

Multivariable logistic regression model showing the association between sociodemographic risk factors and hospitalization among all notified dengue cases in Valle del Cauca in 2022. Hosmer-Lemeshow p=0.40. Link-test squared term p=0.15. OR, Odds Ratio; CI, Confidence Interval. Reference categories: socioeconomic strata 3–6; male sex; other ethnicity (Other, Indigenous, ROM/Gitano, Raizal, and Palenquero); contributory insurance; and age 20–29 years.

^1^Missing values stratum: n = 385 (9.7% of total cases).
