# Supplementary material for: Spatio-temporal clustering of dengue in Valle del Cauca, Colombia
Source: PLoS Negl Trop Dis. 2026 Nov 10;20(11):e0014855. doi: 10.1371/journal.pntd.0014855 (PMC13766613; doi:10.1371/journal.pntd.0014855)
Supplement: Supplementary file 6 [file pntd.0014855.s002.docx]

**S2 Table. Demographic and Clinical Characteristics of Patients with Dengue by Laboratory Confirmation Status in Valle del Cauca, Colombia, 2022, N = 3,964**

| **Characteristic** | **Non-Laboratory Confirmed (N=1,732)** | **Laboratory Confirmed (N=2,232)** | **P-value** |
| --- | --- | --- | --- |
| **Age (years)** |  |  | **<0.001** |
| Median (IQR) | 24 (11-38) | 16 (8-36) |  |
| **Sex** |  |  | **0.342** |
| Male | 935 (54.0%) | 1,171 (52.5%) |  |
| Female | 797 (46.0%) | 1,061 (47.5%) |  |
| **Ethnicity** |  |  | **0.055** |
| Black/Afro-Colombian | 60 (3.5%) | 54 (2.4%) |  |
| Other^1^ | 1,672 (96.5%) | 2,178 (97.6%) |  |
| **SES**^2^ |  |  | **<0.001** |
| 1: Low-Low | 349 (20.2%) | 309 (13.8%) |  |
| 2: Low | 733 (42.3%) | 939 (42.1%) |  |
| 3: Low-Medium | 313 (18.1%) | 614 (27.5%) |  |
| 4: Medium | 41 (2.4%) | 164 (7.3%) |  |
| 5: Medium-High | 28 (1.6%) | 58 (2.6%) |  |
| 6: High | 11 (0.6%) | 20 (0.9%) |  |
| **Severity of Disease** |  |  | **<0.001** |
| Without warning signs | 1,217 (70.3%) | 1,311 (58.7%) |  |
| With warning signs | 508 (29.3%) | 878 (39.3%) |  |
| Severe dengue | 7 (0.4%) | 43 (1.9%) |  |
| **Clinical care setting** |  |  | **<0.001** |
| Ambulatory | 1,034 (59.7%) | 1,095 (49.1%) |  |
| General floor | 269 (15.5%) | 785 (35.2%) |  |
| ICU | 14 (0.8%) | 103 (4.6%) |  |
| Observation | 166 (9.6%) | 178 (8.0%) |  |
| Referral to hospital | 249 (14.4%) | 71 (3.2%) |  |
| **Hospitalization** |  |  | **<0.001** |
| Yes | 351 (20.3%) | 1028 (46.1%) |  |
| No | 1381 (79.7%) | 1204 (53.9%) |  |
| **Insurance Status** |  |  | **<0.001** |
| No insurance | 23 (1.3%) | 37 (1.7%) |  |
| Contributory | 1,189 (68.6%) | 1,542 (69.1%) |  |
| Subsidized | 497 (28.7%) | 579 (25.9%) |  |
| Other | 23 (1.3%) | 74 (3.3%) |  |

Demographic and clinical characteristics of patients by laboratory confirmation group among dengue cases in Valle del Cauca, Colombia, 2022. Bivariate analyses: Wilcoxon-Rank for age. Fisher exact test for ethnicity. All others: Chi-square test.

^1^Other, Indigenous, ROM/Gitano, Raizal, Palenquero.

^2^Missing values stratum: n=385 (9.7%)
