# Supplementary material for: Spatio-temporal clustering of dengue in Valle del Cauca, Colombia
Source: PLoS Negl Trop Dis. 2026 Nov 10;20(11):e0014855. doi: 10.1371/journal.pntd.0014855 (PMC13766613; doi:10.1371/journal.pntd.0014855)
Supplement: Supplementary file 7 [file pntd.0014855.s003.docx]

**S3 Table. Sensitivity of the dengue space-time scan to maximum temporal windows of 1, 3, and 6 months in Valle del Cauca, Colombia, 2022.**

| **Measure** | **6 months** | **3 months** | **1 month** |
| --- | --- | --- | --- |
| Cases analyzed | 1,973 | 1,973 | 1,973 |
| Significant clusters | 28 | 18 | 13 |
| Unique cases within clusters, n (%) | 632 (32.0) | 444 (22.5) | 190 (9.6) |
| Cluster duration, median (range), months | 6 (1–6) | 3 (2–3) | 1 (1–1) |
| Clusters reaching duration cap, n (%) | 20 (71.4) | 15 (83.3) | 13 (100.0) |
| Cluster RR, median | 14.97 | 8.99 | 18.60 |
| Cluster radius, range, km | 0–0.98 | 0–0.98 | 0–0.95 |
| Warm–dry months only, n | 1 | 5 | 7 |
| Cool–wet months only, n | 1 | 2 | 6 |
| Both seasonal categories, n | 26 | 11 | 0 |
| Clusters by municipality |  |  |  |
| Cali | 23 | 17 | 13 |
| Palmira | 1 | 0 | 0 |
| Yumbo | 1 | 1 | 0 |
| Jamundí | 1 | 0 | 0 |
| Buga | 0 | 0 | 0 |
| Tuluá | 1 | 0 | 0 |
| Florida | 1 | 0 | 0 |
| Cartago | 0 | 0 | 0 |
| Candelaria | NA | NA | NA |

Scans used 1,973 cases, 20% maximum population window, 1 km maximum radius, minimum 10 cases, monthly time units, 9,999 Monte Carlo replications, and p < 0.05. Six-month scan represents the primary analysis. Warm–dry months: December–February and June–September, cool–wet months: March–May and October–November. Candelaria had no eligible laboratory-confirmed cases within the study area and was not scanned. Single-cell clusters have a SaTScan radius of zero. The population raster resolution is approximately 100 × 100 m. RR, relative risk; NA, not applicable.
