# Supplementary material for: Spatio-temporal clustering of dengue in Valle del Cauca, Colombia
Source: PLoS Negl Trop Dis. 2026 Nov 10;20(11):e0014855. doi: 10.1371/journal.pntd.0014855 (PMC13766613; doi:10.1371/journal.pntd.0014855)
Supplement: Peer review history

# Peer review: Decision Letter 0

**DOI:** 10.1371/journal.pntd.0014855.r001

**Authors:** Helton Santiago, Mabel Carabali

24 Mar 2026

PNTD-D-26-00122Spatio-temporal clustering of dengue in Valle del Cauca, ColombiaPLOS Neglected Tropical DiseasesDear Dr. Sy,Thank you for submitting your manuscript to PLOS Neglected Tropical Diseases. After careful consideration, we feel that it has merit but does not fully meet PLOS Neglected Tropical Diseases's publication criteria as it currently stands. Therefore, we invite you to submit a revised version of the manuscript that addresses the points raised during the review process.Please submit your revised manuscript by May 23 2026 11:59PM. If you will need more time than this to complete your revisions, please reply to this message or contact the journal office at plosntds@plos.org. When you're ready to submit your revision, log on to https://www.editorialmanager.com/pntd/ and select the 'Submissions Needing Revision' folder to locate your manuscript file.Please include the following items when submitting your revised manuscript:* A letter that responds to each point raised by the editor and reviewer(s). You should upload this letter as a separate file labeled 'Response to Reviewers'. This file does not need to include responses to any formatting updates and technical items listed in the 'Journal Requirements' section below.* A marked-up copy of your manuscript that highlights changes made to the original version. You should upload this as a separate file labeled 'Revised Manuscript with Track Changes'.* An unmarked version of your revised paper without tracked changes. You should upload this as a separate file labeled 'Manuscript'.If you would like to make changes to your financial disclosure, competing interests statement, or data availability statement, please make these updates within the submission form at the time of resubmission. Guidelines for resubmitting your figure files are available below the reviewer comments at the end of this letter.We look forward to receiving your revised manuscript.Kind regards,Helton C. Santiago, M.D., Ph.DAcademic EditorPLOS Neglected Tropical DiseasesMabel CarabaliSection EditorPLOS Neglected Tropical Diseases

Shaden Kamhawi

co-Editor-in-Chief

PLOS Neglected Tropical Diseases

orcid.org/0000-0003-4304-636XX

Paul Brindley

co-Editor-in-Chief

PLOS Neglected Tropical Diseases

orcid.org/0000-0003-1765-0002

**Additional Editor Comments:** The manuscript was evaluated by three experts in the field and considered of interest. Reviewers recommended minor revision. Please, address the comments as fully as possible. Be aware of the attached comments.**Journal Requirements:**

1) Please ensure that the CRediT author contributions listed for every co-author are completed accurately and in full.

At this stage, the following Authors/Authors require contributions: Hendrik Sy, Deninson Alejandro Vargas-Gonzalez, Freddy Córdoba-Rentaría, Albert Icksang Ko, Johanna Patricia Daily, and Neal Douglas Alexander. Please ensure that the full contributions of each author are acknowledged in the "Add/Edit/Remove Authors" section of our submission form.

The list of CRediT author contributions may be found here: https://journals.plos.org/plosntds/s/authorship#loc-author-contributions

2) We ask that a manuscript source file is provided at Revision. Please upload your manuscript file as a .doc, .docx, .rtf or .tex. If you are providing a .tex file, please upload it under the item type u2018LaTeX Source Fileu2019 and leave your .pdf version as the item type u2018Manuscriptu2019.

3) We do not publish any copyright or trademark symbols that usually accompany proprietary names, eg ©,  ®, or TM  (e.g. next to drug or reagent names). Therefore please remove all instances of trademark/copyright symbols throughout the text, including:

- © on page: 6 and 14.

- ® on page: 6..

4) Please upload all main figures as separate Figure files in .tif or .eps format. For more information about how to convert and format your figure files please see our guidelines:

https://journals.plos.org/plosntds/s/figures

5) We notice that your supplementary Tables are included in the manuscript file. Please remove them and upload them with the file type 'Supporting Information'. Please ensure that each Supporting Information file has a legend listed in the manuscript after the references list.

6) In the online submission form, you indicated that "The data underlying the results presented in the study are available from the Secretaría de Salud del Valle del Cauca. Contact: contactenos@valledelcauca.gov.co". All PLOS journals now require all data underlying the findings described in their manuscript to be freely available to other researchers, either

1. In a public repository

2. Within the manuscript itself

3. Uploaded as supplementary information.

This policy applies to all data except where public deposition would breach compliance with the protocol approved by your research ethics board. If your data cannot be made publicly available for ethical or legal reasons (e.g., public availability would compromise patient privacy), please explain your reasons by return email and your exemption request will be escalated to the editor for approval. Your exemption request will be handled independently and will not hold up the peer review process, but will need to be resolved should your manuscript be accepted for publication. One of the Editorial team will then be in touch if there are any issues.

**Reviewers' comments:** Reviewer's Responses to Questions

**Key Review Criteria Required for Acceptance?**

As you describe the new analyses required for acceptance, please consider the following:

**Methods**

-Are the objectives of the study clearly articulated with a clear testable hypothesis stated?

-Is the study design appropriate to address the stated objectives?

-Is the population clearly described and appropriate for the hypothesis being tested?

-Is the sample size sufficient to ensure adequate power to address the hypothesis being tested?

-Were correct statistical analysis used to support conclusions?

-Are there concerns about ethical or regulatory requirements being met?

Reviewer #1: The methods are well described, but some aspects need to be clarified. Please see attachment

Reviewer #2: see report

Reviewer #3: -Are the objectives of the study clearly articulated with a clear testable hypothesis stated?

The objectives are clearly stated: to identify sub-municipal spatio-temporal clusters of laboratory-confirmed dengue cases and to assess factors associated with hospitalization. However, a formal testable hypothesis is not explicitly stated.

-Is the study design appropriate to address the stated objectives?

Yes. The retrospective observational design is appropriate for both the cluster analysis and the hospitalization risk analysis. The use of surveillance data, geocoded residences, SaTScan, and multivariable logistic regression is suitable for the stated aims.

-Is the population clearly described and appropriate for the hypothesis being tested?

Yes. The population is clearly described and appropriate. The spatial analysis included laboratory-confirmed cases from nine high-risk municipalities, and the hospitalization analysis included laboratory-confirmed dengue cases from Valle del Cauca reported in 2022.

-Is the sample size sufficient to ensure adequate power to address the hypothesis being tested?

Yes for the hospitalization analysis. The authors performed a power calculation and the final analytical sample exceeded the required sample size for 80% power.

-Were correct statistical analyses used to support conclusions?

Overall, yes. The use of Kulldorff’s space-time scan statistic and multivariable logistic regression is appropriate. The manuscript also includes assessment of confounding, multicollinearity, model fit, and a sensitivity analysis.

-Are there concerns about ethical or regulatory requirements being met?

No major concerns. The study reports IRB approvals and compliance with international and Colombian ethical guidelines.

**********

**Results**

-Does the analysis presented match the analysis plan?

-Are the results clearly and completely presented?

-Are the figures (Tables, Images) of sufficient quality for clarity?

Reviewer #1: Well presented!

Reviewer #2: see report

Reviewer #3: -Does the analysis presented match the analysis plan?

Overall, yes. The Results follow the two stated aims and the Methods: first, the spatio-temporal cluster analysis, and second, the hospitalization risk analysis. The manuscript reports the number, size, timing, and distribution of clusters, and then presents the bivariate, multivariable, and sensitivity analyses for hospitalization, which is consistent with the analytical approach described earlier.

-Are the results clearly and completely presented? Yes, overall. The results are logically organized, clearly separated by objective, and the main findings are reported with appropriate numerical detail. The presentation of cluster counts, timing, and proportion of cases within clusters is clear, and the hospitalization analysis reports effect estimates with confidence intervals and p-values. A few minor editorial issues remain. For example, there is an incomplete parenthesis in the sentence reporting hospitalization frequency by SES group, and some table formatting is slightly rough in the draft. These are small presentation issues rather than substantive problems.

- Are the figures (Tables, Images) of sufficient quality for clarity? Yes, in general. The flow chart, cluster table, maps, epidemic curve, and regression tables are all relevant and help the reader understand the findings. Figure 3 is particularly useful for visualizing the focal nature of clustering in Cali, and Figure 4 effectively summarizes timing and duration of clusters across municipalities. Tables 3 and 4 are also informative and readable.

- My only minor suggestion is to improve final formatting and resolution where possible, especially in the map and table presentation, to ensure optimal readability in publication format.

**********

**Conclusions**

-Are the conclusions supported by the data presented?

-Are the limitations of analysis clearly described?

-Do the authors discuss how these data can be helpful to advance our understanding of the topic under study?

-Is public health relevance addressed?

Reviewer #1: The limitations section should be supported with more evidence. See attachment

Reviewer #2: see report

Reviewer #3: -Are the conclusions supported by the data presented?

Yes, overall. The conclusions are consistent with the main findings: the study identified 35 sub-municipal dengue clusters, with 45% of laboratory-confirmed cases falling within clusters, and found higher odds of hospitalization among patients with low SES, subsidized or no insurance, and older age. The claims are appropriately framed and not overstated.

-Are the limitations of analysis clearly described?

Yes. The authors acknowledge important limitations, including the circular scan window, imperfect population estimates, reliance on passive surveillance data, missing data, possible reporting errors, and the use of residential address as a proxy for infection site. They also note that human mobility and other socio-environmental determinants were not captured.

-Do the authors discuss how these data can be helpful to advance our understanding of the topic under study?

Yes. The manuscript shows that dengue transmission in Valle del Cauca is focal at the sub-municipal level and may persist across seasons, which adds useful operational knowledge for outbreak response. It also highlights possible social disparities in hospitalization and suggests directions for future research, including integration of climate, entomological, and intervention data across multiple years.

-Is public health relevance addressed?

Yes, clearly. The authors explicitly link their findings to reactive vector control, prioritization of limited resources, and the need for equitable healthcare access for vulnerable populations, particularly low-income groups and older adults. This public health relevance is one of the manuscript’s main strengths.

**********

**Editorial and Data Presentation Modifications?**

Use this section for editorial suggestions as well as relatively minor modifications of existing data that would enhance clarity. If the only modifications needed are minor and/or editorial, you may wish to recommend “Minor Revision” or “Accept”.

Reviewer #1: Minor Revision

Reviewer #2: see report

Reviewer #3: "Minor Revision"

The manuscript is strong overall and only minor editorial and presentation-related revisions are needed. I suggest careful copyediting for grammar, punctuation, and consistency of style throughout the text. A few examples include subject–verb agreement in the Introduction (“targeted reactive interventions … requires”), typographical issues in figure legends (e.g., “municipalitas” in Figure 1), and some incomplete or awkwardly punctuated sentences in the Results section, including the sentence reporting hospitalization frequency by SES group.

There are also several minor formatting issues that should be corrected for clarity. In the abstract, the sentence ending with the hospitalization findings appears to stop abruptly with a trailing comma. In the Results, there is a stray character before the paragraph introducing Table 3, and some table footnotes and percentages should be standardized, for example the decimal notation in “3,2%” and the presentation of percentages in supplementary tables.

The tables and figures are informative, but their presentation could be refined slightly to improve readability in the final published version. Table 1 would benefit from minor formatting cleanup to improve alignment and readability of municipality names and summary measures, and the map/cluster figures should be checked to ensure optimal resolution and legibility of labels in publication format.

Finally, the reference list and data availability text would benefit from a final editorial check for consistency in web citations, access-date formatting, and typographical accuracy. For example, “Februay” should be corrected, “Avaiable” should be corrected, and repeated punctuation in some web references should be standardized. The data availability statement may also need to be aligned carefully with journal policy language.

Overall, these are minor issues that do not affect the scientific value of the study, and I would support publication after minor revision.

**********

**Summary and General Comments**

Use this section to provide overall comments, discuss strengths/weaknesses of the study, novelty, significance, general execution and scholarship. You may also include additional comments for the author, including concerns about dual publication, research ethics, or publication ethics. If requesting major revision, please articulate the new experiments that are needed.

Reviewer #1: The authors present an important study that can inform the targeting of future public health interventions, using standard methodological approaches. The manuscript is well written and easy to follow. Nevertheless, several methodological and analytical aspects require clarification.

Reviewer #2: see report

Reviewer #3: The conclusions are supported by the data and are appropriately linked to the study objectives. The limitations are clearly acknowledged, and the manuscript makes a useful contribution by showing how sub-municipal clustering analysis may help guide reactive dengue control and resource allocation. The public health relevance is clear, particularly regarding targeted interventions and possible inequities in dengue hospitalization.

**********

PLOS authors have the option to publish the peer review history of their article (what does this mean?). If published, this will include your full peer review and any attached files.

If you choose “no”, your identity will remain anonymous but your review may still be made public.

**Do you want your identity to be public for this peer review?**  For information about this choice, including consent withdrawal, please see our Privacy Policy.

Reviewer #1: No

Reviewer #2: No

Reviewer #3: **Yes:**  Rocio Cardenas

[NOTE: If reviewer comments were submitted as an attachment file, they will be attached to this email and accessible via the submission site. Please log into your account, locate the manuscript record, and check for the action link "View Attachments". If this link does not appear, there are no attachment files.] **Figure resubmission:** While revising your submission, we strongly recommend that you use PLOS’s NAAS tool (https://ngplosjournals.pagemajik.ai/artanalysis) to test your figure files. NAAS can convert your figure files to the TIFF file type and meet basic requirements (such as print size, resolution), or provide you with a report on issues that do not meet our requirements and that NAAS cannot fix.

After uploading your figures to PLOS’s NAAS tool - https://ngplosjournals.pagemajik.ai/artanalysis, NAAS will process the files provided and display the results in the "Uploaded Files" section of the page as the processing is complete. If the uploaded figures meet our requirements (or NAAS is able to fix the files to meet our requirements), the figure will be marked as "fixed" above. If NAAS is unable to fix the files, a red "failed" label will appear above. When NAAS has confirmed that the figure files meet our requirements, please download the file via the download option, and include these NAAS processed figure files when submitting your revised manuscript.**Reproducibility:** To enhance the reproducibility of your results, we recommend that authors of applicable studies deposit laboratory protocols in protocols.io, where a protocol can be assigned its own identifier (DOI) such that it can be cited independently in the future. Additionally, PLOS ONE offers an option to publish peer-reviewed clinical study protocols. Read more information on sharing protocols at https://plos.org/protocols?utm_medium=editorial-email&utm_source=authorletters&utm_campaign=protocols

Attachment Submitted filename: Reviewer PLOS.pdf

Attachment Submitted filename: REVIEWS-1.pdf

# Author comment: Author response to Decision Letter 1

**DOI:** 10.1371/journal.pntd.0014855.r002

1 Jul 2026

Attachment Submitted filename: Response to Reviewers.pdf

# Peer review: Decision Letter 1

**DOI:** 10.1371/journal.pntd.0014855.r003

**Authors:** Helton Santiago, Michael Holbrook

30 Jul 2026

PNTD-D-26-00122R1Spatio-temporal clustering of dengue in Valle del Cauca, ColombiaPLOS Neglected Tropical Diseases Dear Dr. Sy, Thank you for submitting your manuscript to PLOS Neglected Tropical Diseases. After careful consideration, we feel that it has merit but does not fully meet PLOS Neglected Tropical Diseases's publication criteria as it currently stands. Therefore, we invite you to submit a revised version of the manuscript that addresses the points raised during the review process. Please submit your revised manuscript by Sep 28 2026 11:59PM. If you will need more time than this to complete your revisions, please reply to this message or contact the journal office at plosntds@plos.org. When you're ready to submit your revision, log on to https://www.editorialmanager.com/pntd/ and select the 'Submissions Needing Revision' folder to locate your manuscript file. Please include the following items when submitting your revised manuscript:* A letter that responds to each point raised by the editor and reviewer(s). You should upload this letter as a separate file labeled 'Response to Reviewers'. This file does not need to include responses to any formatting updates and technical items listed in the 'Journal Requirements' section below.* A marked-up copy of your manuscript that highlights changes made to the original version. You should upload this as a separate file labeled 'Revised Manuscript with Track Changes'.* An unmarked version of your revised paper without tracked changes. You should upload this as a separate file labeled 'Manuscript'. If you would like to make changes to your financial disclosure, competing interests statement, or data availability statement, please make these updates within the submission form at the time of resubmission. Guidelines for resubmitting your figure files are available below the reviewer comments at the end of this letter. As the corresponding author, your ORCID iD is verified in the submission system and will appear in the published article. PLOS supports the use of ORCID, and we encourage all coauthors to register for an ORCID iD and use it as well. Please encourage your coauthors to verify their ORCID iD within the submission system before final acceptance, as unverified ORCID iDs will not appear in the published article. Only the individual author can complete the verification step; PLOS staff cannot verify ORCID iDs on behalf of authors.We look forward to receiving your revised manuscript. Kind regards, Helton C. Santiago, M.D., Ph.DAcademic EditorPLOS Neglected Tropical Diseases Michael HolbrookSection EditorPLOS Neglected Tropical Diseases

Shaden Kamhawi

co-Editor-in-Chief

PLOS Neglected Tropical Diseases

orcid.org/0000-0003-4304-636XX

Paul Brindley

co-Editor-in-Chief

PLOS Neglected Tropical Diseases

orcid.org/0000-0003-1765-0002

**Additional Editor Comments (if provided):**  The editors and reviewers acknowledge the improvements in the manuscript with the revision process However, two reviewers still identified minor problems that should be addressed before acceptance. Please, address them and return as soon as possible. **Reviewers' comments:**  Reviewer's Responses to Questions

**Key Review Criteria Required for Acceptance?**

As you describe the new analyses required for acceptance, please consider the following:

**Methods**

-Are the objectives of the study clearly articulated with a clear testable hypothesis stated?

-Is the study design appropriate to address the stated objectives?

-Is the population clearly described and appropriate for the hypothesis being tested?

-Is the sample size sufficient to ensure adequate power to address the hypothesis being tested?

-Were correct statistical analysis used to support conclusions?

-Are there concerns about ethical or regulatory requirements being met?

Reviewer #1: The study clearly states two main objectives: to identify spatio-temporal clusters of laboratory-confirmed dengue cases at the sub-municipal level and to assess associations between sociodemographic and clinical characteristics and dengue-related hospitalization. The retrospective observational design using SIVIGILA surveillance data is appropriate, and the study population, eligibility criteria, and selected municipalities are well described. The sample size appears sufficient, particularly for the hospitalization analysis, as the final sample exceeded the required number estimated in the power calculation. The statistical methods, including Kulldorff's space-time scan statistic and multivariable logistic regression, are appropriate and supported by sensitivity analyses and model diagnostics.

Reviewer #2: Yes, all is sound. But I still do not believe that 50% is an ok parameter in Satscan for an infectious diseases. If it corresponds up to six month, that is too long.

Reviewer #3: Yes. The authors have adequately addressed this comment by adding an explicit and testable hypothesis aligned with both study aims. No further revision is required.

**********

**Results**

-Does the analysis presented match the analysis plan?

-Are the results clearly and completely presented?

-Are the figures (Tables, Images) of sufficient quality for clarity?

Reviewer #1: The analyses presented in the Results section are consistent with the stated analysis plan. Overall, the results are clearly and adequately presented. The figures and tables are of sufficient quality and facilitate the interpretation of the main findings. The tables summarize the results of the analyses examining factors associated with hospitalization.

Reviewer #2: The map with the clusters needs work.

The figure is visually overcrowded, making it difficult for the reader to identify the main spatial patterns. The large number of crossing leader lines, overlapping proportional symbols, and repeated text labels substantially reduces readability. I recommend redesigning the figure to improve visual hierarchy by minimizing leader lines, simplifying the labeling strategy, reducing redundancy in the annotations, and considering alternative approaches (e.g., direct labeling, separate panels for temporal information, or a companion table). These changes would greatly improve the clarity and interpretability of the map.

Reviewer #3: The analysis presented generally matches the analysis plan and the Results are organized consistently with the two stated aims. The revised cluster counts appropriately reflect the analysis using 9,999 Monte Carlo replications, and the hospitalization analysis and sensitivity analyses are presented in a logical sequence.

The Results are largely clear and complete. However, two minor reporting issues should be addressed before acceptance. First, in Table 3, the estimate for female sex is reported as OR 1.19 (95% CI 0.94–1.34), with p=0.21. The confidence interval and p-value do not appear internally consistent and should be verified against the original regression output. Second, the statement that “a significant interaction was observed between hospitalization and dengue severity” is not statistically clear because hospitalization is the dependent variable. The authors should specify the actual interaction term evaluated or revise this sentence.

Figure quality has improved, but Figure 3 remains visually crowded, with several overlapping labels and leader lines that reduce readability. Figure 4 also appears partially truncated in the compiled reviewer PDF. The authors or production team should verify that the complete figure, axes, time scale, municipality labels, and annotations are clearly visible in the final files.

These are minor reporting and presentation issues and do not alter the overall interpretation or conclusions of the study.

**********

**Conclusions**

-Are the conclusions supported by the data presented?

-Are the limitations of analysis clearly described?

-Do the authors discuss how these data can be helpful to advance our understanding of the topic under study?

-Is public health relevance addressed?

Reviewer #1: The conclusions are well supported by the data presented and are consistent with the main findings of the study. The identification of localized spatio-temporal dengue clusters may provide useful information for health authorities to better prioritize and target future surveillance and vector-control activities. The limitations of the analyses are clearly acknowledged and discussed. The authors also explain how these findings contribute to a better understanding of dengue transmission at the sub-municipal level. The study has clear public health relevance, particularly in low- and middle-income settings where resources are limited and more precise identification of high-risk areas could support a more efficient allocation of prevention and control resources.

Reviewer #2: (No Response)

Reviewer #3: The conclusions are generally supported by the data presented. The authors appropriately state that the identified sub-municipal clusters may help inform reactive vector-control activities, and the main Conclusions use cautious language when interpreting the association between socioeconomic status and hospitalization.

The limitations are clearly and comprehensively described. The revised manuscript acknowledges the constraints of circular scan windows, the use of modeled WorldPop denominators, sensitivity to the selected spatial and temporal parameters, passive-surveillance underreporting, diagnostic-ascertainment bias, missing data, the socioeconomic-stratum grouping, residential address as a proxy for infection location, and the absence of mobility and other socio-environmental variables.

The manuscript also explains how the findings may advance understanding of dengue transmission by showing that reported cases may cluster at a small sub-municipal scale and persist across seasonal periods. The potential operational value for prioritizing limited vector-control resources and identifying populations with higher odds of hospitalization is clearly discussed, and the public health relevance is adequately addressed.

**********

**Editorial and Data Presentation Modifications?**

Use this section for editorial suggestions as well as relatively minor modifications of existing data that would enhance clarity. If the only modifications needed are minor and/or editorial, you may wish to recommend “Minor Revision” or “Accept”.

Reviewer #1: No additional editorial or data presentation modifications are suggested.

Reviewer #2: (No Response)

Reviewer #3: The authors have satisfactorily addressed most of the previous editorial and data-presentation comments. Only a limited number of minor corrections remain:

1-Table 3: Please verify the estimate reported for female sex. The OR of 1.19, 95% CI of 0.94–1.34, and p=0.21 do not appear fully internally consistent. The values should be checked against the original regression output.

Reference categories in Table 3: Please state explicitly the reference categories for socioeconomic stratum, sex, ethnicity, insurance status, and age, either within the table or in a footnote. At present, some reference groups are only implied.

2-Interaction statement: The sentence stating that “a significant interaction was observed between hospitalization and dengue severity” should be clarified. Hospitalization is the dependent outcome and therefore should not be described as one component of an interaction term. Please specify the actual interaction evaluated or revise the sentence.

3-Figure 3: Although the resolution has improved, the map remains crowded because several cluster labels and leader lines overlap. Minor repositioning or simplification of the labels would improve readability.

4-Figure 4: Please verify the final uploaded figure and compiled proof, as the right side appears partially truncated in the reviewer PDF. The complete time axis, municipality labels, cluster bars, and seasonal shading should remain visible.

5-Discussion grammar: The sentence beginning “Our findings suggest that among patients with dengue…” should be revised for subject–verb agreement and clarity. For example: “Our findings suggest that lower socioeconomic status, lack of insurance, and enrollment in the state-subsidized insurance scheme were associated with higher odds of hospitalization.”

6-Data availability: The statement should specify that access to de-identified data is subject to institutional review and a data-use agreement, as indicated in the response letter. The authors should also clarify whether the analytical code can be made publicly available, since it would not contain individual-level identifiable information.

**********

**Summary and General Comments**

Use this section to provide overall comments, discuss strengths/weaknesses of the study, novelty, significance, general execution and scholarship. You may also include additional comments for the author, including concerns about dual publication, research ethics, or publication ethics. If requesting major revision, please articulate the new experiments that are needed.

Reviewer #1: Overall, the manuscript is clearly written, well structured, and easy to follow. The authors address an important public health question and use an appropriate study design and analytical approach to identify spatio-temporal dengue clusters and explore factors associated with hospitalization. The study objectives are clearly defined and adequately addressed by the analyses presented. A particular strength of the study is the identification of localized high-risk areas that could help public health authorities better prioritize surveillance and design more targeted vector-control interventions.

Reviewer #2: (No Response)

Reviewer #3: This is a relevant and well-executed study addressing the spatial and temporal concentration of dengue cases at a scale that may be useful for local public health action. The manuscript combines two complementary components: detection of sub-municipal spatio-temporal clusters of laboratory-confirmed dengue cases and assessment of sociodemographic factors associated with hospitalization. This combination strengthens the applied value of the work.

The principal contribution is operational rather than methodological. The study does not introduce a new spatial-statistical method, but it applies an established space-time scan statistic using stakeholder-informed spatial constraints intended to generate intervention areas that are potentially manageable by local health authorities. The restriction to laboratory-confirmed cases, the sub-kilometer cluster scale, and the linkage with patient-level hospitalization analysis distinguish the study from some previous spatial analyses in Cali and Colombia. The manuscript is generally clearly written, appropriately referenced, and scientifically sound. The conclusions are mostly proportional to the evidence, and the public health relevance is well articulated. The authors have responded constructively to the previous reviews and have substantially strengthened the manuscript. The remaining concerns are limited to minor issues of statistical reporting, wording, figure readability, and editorial clarity, as detailed in the preceding sections. I therefore recommend Minor Revision.

**********

PLOS authors have the option to publish the peer review history of their article (what does this mean?). If published, this will include your full peer review and any attached files.

If you choose “no”, your identity will remain anonymous but your review may still be made public.

**Do you want your identity to be public for this peer review?**  For information about this choice, including consent withdrawal, please see our Privacy Policy.

Reviewer #1: **Yes:**  Maria Angelica Carrillo

Reviewer #2: No

Reviewer #3: No

[NOTE: If reviewer comments were submitted as an attachment file, they will be attached to this email and accessible via the submission site. Please log into your account, locate the manuscript record, and check for the action link "View Attachments". If this link does not appear, there are no attachment files.] **Figure resubmission:**   While revising your submission, we strongly recommend that you use PLOS’s NAAS tool (https://ngplosjournals.pagemajik.ai/artanalysis) to test your figure files. NAAS can convert your figure files to the TIFF file type and meet basic requirements (such as print size, resolution), or provide you with a report on issues that do not meet our requirements and that NAAS cannot fix.

After uploading your figures to PLOS’s NAAS tool - https://ngplosjournals.pagemajik.ai/artanalysis, NAAS will process the files provided and display the results in the "Uploaded Files" section of the page as the processing is complete. If the uploaded figures meet our requirements (or NAAS is able to fix the files to meet our requirements), the figure will be marked as "fixed" above. If NAAS is unable to fix the files, a red "failed" label will appear above. When NAAS has confirmed that the figure files meet our requirements, please download the file via the download option, and include these NAAS processed figure files when submitting your revised manuscript. **Reproducibility:**  To enhance the reproducibility of your results, we recommend that authors of applicable studies deposit laboratory protocols in protocols.io, where a protocol can be assigned its own identifier (DOI) such that it can be cited independently in the future. Additionally, PLOS ONE offers an option to publish peer-reviewed clinical study protocols. Read more information on sharing protocols at https://plos.org/protocols?utm_medium=editorial-email&utm_source=authorletters&utm_campaign=protocols

# Author comment: Author response to Decision Letter 2

**DOI:** 10.1371/journal.pntd.0014855.r004

14 Oct 2026

Attachment Submitted filename: Response_to_Reviewers_auresp_2.pdf

# Peer review: Decision Letter 2

**DOI:** 10.1371/journal.pntd.0014855.r005

**Authors:** Helton Santiago, Michael Holbrook

25 Oct 2026

Dear Sy,

We are pleased to inform you that your manuscript 'Spatio-temporal clustering of dengue in Valle del Cauca, Colombia' has been provisionally accepted for publication in PLOS Neglected Tropical Diseases.

Before your manuscript can be formally accepted you will need to complete some formatting changes, which you will receive in a follow up email. A member of our team will be in touch with a set of requests.

Please note that your manuscript will not be scheduled for publication until you have made the required changes, so a swift response is appreciated.

IMPORTANT: The editorial review process is now complete. PLOS will only permit corrections to spelling, formatting or significant scientific errors from this point onwards. Requests for major changes, or any which affect the scientific understanding of your work, will cause delays to the publication date of your manuscript.

Should you, your institution's press office or the journal office choose to press release your paper, you will automatically be opted out of early publication. We ask that you notify us now if you or your institution is planning to press release the article. All press must be co-ordinated with PLOS.

Thank you again for supporting Open Access publishing; we are looking forward to publishing your work in PLOS Neglected Tropical Diseases.

Best regards,

Helton C. Santiago, M.D., Ph.D

Academic Editor

PLOS Neglected Tropical Diseases

Michael Holbrook

Section Editor

PLOS Neglected Tropical Diseases

Shaden Kamhawi

co-Editor-in-Chief

PLOS Neglected Tropical Diseases

orcid.org/0000-0003-4304-636XX

Paul Brindley

co-Editor-in-Chief

PLOS Neglected Tropical Diseases

orcid.org/0000-0003-1765-0002

***********************************************************

Reviewer's Responses to Questions

**Key Review Criteria Required for Acceptance?**

As you describe the new analyses required for acceptance, please consider the following:

**Methods**

-Are the objectives of the study clearly articulated with a clear testable hypothesis stated?

-Is the study design appropriate to address the stated objectives?

-Is the population clearly described and appropriate for the hypothesis being tested?

-Is the sample size sufficient to ensure adequate power to address the hypothesis being tested?

-Were correct statistical analysis used to support conclusions?

-Are there concerns about ethical or regulatory requirements being met?

Reviewer #2: (No Response)

**********

**Results**

-Does the analysis presented match the analysis plan?

-Are the results clearly and completely presented?

-Are the figures (Tables, Images) of sufficient quality for clarity?

Reviewer #2: (No Response)

**********

**Conclusions**

-Are the conclusions supported by the data presented?

-Are the limitations of analysis clearly described?

-Do the authors discuss how these data can be helpful to advance our understanding of the topic under study?

-Is public health relevance addressed?

Reviewer #2: (No Response)

**********

**Editorial and Data Presentation Modifications?**

Use this section for editorial suggestions as well as relatively minor modifications of existing data that would enhance clarity. If the only modifications needed are minor and/or editorial, you may wish to recommend “Minor Revision” or “Accept”.

Reviewer #2: (No Response)

**********

**Summary and General Comments**

Use this section to provide overall comments, discuss strengths/weaknesses of the study, novelty, significance, general execution and scholarship. You may also include additional comments for the author, including concerns about dual publication, research ethics, or publication ethics. If requesting major revision, please articulate the new experiments that are needed.

Reviewer #2: (No Response)

**********

PLOS authors have the option to publish the peer review history of their article (what does this mean?). If published, this will include your full peer review and any attached files.

If you choose “no”, your identity will remain anonymous but your review may still be made public.

**Do you want your identity to be public for this peer review?**  For information about this choice, including consent withdrawal, please see our Privacy Policy.

Reviewer #2: No

# Editor report: Acceptance letter

**DOI:** 10.1371/journal.pntd.0014855.r006

**Authors:** Helton Santiago, Michael Holbrook

Dear Sy,

We are delighted to inform you that your manuscript, "Spatio-temporal clustering of dengue in Valle del Cauca, Colombia," has been formally accepted for publication in PLOS Neglected Tropical Diseases.

We have now passed your article onto the PLOS Production Department who will complete the rest of the publication process. All authors will receive a confirmation email upon publication.

The corresponding author will soon be receiving a typeset proof for review, to ensure errors have not been introduced during production. Please review the PDF proof of your manuscript carefully, as this is the last chance to correct any scientific or type-setting errors. Please note that major changes, or those which affect the scientific understanding of the work, will likely cause delays to the publication date of your manuscript. Note: Proofs for Front Matter articles (Editorial, Viewpoint, Symposium, Review, etc...) are generated on a different schedule and may not be made available as quickly.

Soon after your final files are uploaded, the early version of your manuscript will be published online unless you opted out of this process. The date of the early version will be your article's publication date. The final article will be published to the same URL, and all versions of the paper will be accessible to readers.

For Research Articles, you will receive an invoice from PLOS for your publication fee after your manuscript has reached the completed accept phase. If you receive an email requesting payment before acceptance or for any other service, this may be a phishing scheme. Learn how to identify phishing emails and protect your accounts at https://explore.plos.org/phishing.

Thank you again for supporting open-access publishing; we are looking forward to publishing your work in PLOS Neglected Tropical Diseases.

Best regards,

Shaden Kamhawi

co-Editor-in-Chief

PLOS Neglected Tropical Diseases

Paul Brindley

co-Editor-in-Chief

PLOS Neglected Tropical Diseases
